# Coinfection and nonrandom recombination drive the evolution of swine enteric coronaviruses

**DOI:** 10.1080/22221751.2024.2332653

**Published:** 2024-03-22

**Authors:** Jiahui Guo, Yinan Lai, Zhixiang Yang, Wenbo Song, Junwei Zhou, Zhuang Li, Wen Su, Shaobo Xiao, Liurong Fang

**Affiliations:** a National Key Laboratory of Agricultural Microbiology, College of Veterinary Medicine, Huazhong Agricultural University, Wuhan, People’s Republic of China; b Key Laboratory of Preventive Veterinary Medicine in Hubei Province, The Cooperative Innovation Center for Sustainable Pig Production, Wuhan, People’s Republic of China; c Hubei Hongshan Laboratory, Huazhong Agricultural University, Wuhan, People’s Republic of China

**Keywords:** Swine enteric coronaviruses, coinfection, recombination, evolution, epidemiology

## Abstract

Coinfection with multiple viruses is a common phenomenon in clinical settings and is a crucial driver of viral evolution. Although numerous studies have demonstrated viral recombination arising from coinfections of different strains of a specific species, the role of coinfections of different species or genera during viral evolution is rarely investigated. Here, we analyzed coinfections of and recombination events between four different swine enteric coronaviruses that infect the jejunum and ileum in pigs, including porcine epidemic diarrhea virus (PEDV), transmissible gastroenteritis virus (TGEV), and swine acute diarrhea syndrome coronavirus (SADS-CoV), and a deltacoronavirus, porcine deltacoronavirus (PDCoV). Various coinfection patterns were observed in 4,468 fecal and intestinal tissue samples collected from pigs in a 4-year survey. PEDV/PDCoV was the most frequent coinfection. However, recombination analyses have only detected events involving PEDV/TGEV and SADS-CoV/TGEV, indicating that inter-species recombination among coronaviruses is most likely to occur within the same genus. We also analyzed recombination events within the newly identified genus *Deltacoronavirus* and found that sparrows have played a unique host role in the recombination history of the deltacoronaviruses. The emerging virus PDCoV, which can infect humans, has a different recombination history. In summary, our study demonstrates that swine enteric coronaviruses are a valuable model for investigating the relationship between viral coinfection and recombination, which provide new insights into both inter- and intraspecies recombination events among swine enteric coronaviruses, and extend our understanding of the relationship between coronavirus coinfection and recombination.

## Introduction

Coinfection with multiple viruses is a common occurrence in clinical settings, affecting various host species, including humans, livestock, and wildlife [1–3]. Viral coinfections can occur between viruses of distinct families, genera, species, and strains, and exert a significant influence on viral pathogenesis, severity, and the outcomes of infections, exacerbating disease, increasing morbidity and mortality rates, and prolonging recovery periods [2,4]. Coinfection also plays a crucial role in viral evolution, facilitating genetic exchange between viruses of different species and strains, thus increasing the likelihood of recombination and the emergence of novel variants. It potentially contributes to the evolution and emergence of new viral strains with altered pathogenicity and transmissibility. For instance, there is evidence that poliovirus can recombine with other enteroviruses during natural human infections [5,6].

Coronaviruses are a ubiquitous group of viruses that infect mammals and birds. They are classified into four genera: Alphacoronavirus (α-CoV), Betacoronavirus (β-CoV), Gammacoronavirus (γ-CoV), and Deltacoronavirus (δ-CoV) within the family Coronaviridae [7]. The evolution of the coronaviruses is marked by several phenomena, including mutation, recombination, coinfection, and gene loss [8]. Documented cases of coinfections of coronaviruses have involved both human and animal coronaviruses. Examples include coinfections of different swine enteric coronaviruses in piglets with diarrhea [9–12], middle east respiratory syndrome coronavirus (MERS-CoV) has been shown to coinfect and recombine in the lungs of infected dromedary camels, increasing its transmission and potential spillover into human populations, as in the MERS-CoV outbreak in 2015 in Korea [13,14]. Recombinant lineages XD and XE of severe acute respiratory syndrome coronavirus 2 (SARS-CoV-2) were generated through the recombination and coinfection of the SARS-CoV-2 delta and omicron variants and between omicron subvariants BA.1 and BA.2, respectively [15–17].

The genomes of swine enteric coronaviruses are characterized by a large, single-stranded RNA molecule of positive polarity, ranging from 26 to 32 kilobases (kb) in size [18]. It comprises a long open reading frame (ORF) of approximately 20 kb that encodes two polyproteins, pp1a and pp1ab through a frameshift mechanism. Following these polyproteins, there are four additional ORFs, which encode essential conserved proteins: spike (S), envelope (E), matrix (M), and nucleocapsid (N) [19]. The four swine enteric coronaviruses, transmissible gastroenteritis virus (TGEV) [20], porcine epidemic diarrhea virus (PEDV) [21], swine acute diarrhea syndrome coronavirus (SADS-CoV) [22] and porcine deltacoronavirus (PDCoV) [23], share both genetic and structural features and cause severe enteric disease in pigs. They responsible for significant severe diarrhea in pigs in China [23–25]. Coinfection with multiple pathogens poses a challenge for disease control and has attracted increasing attention from researchers [2,3,17,26]. The swine enteric coronaviruses offer a more suitable model for investigating coronaviral coinfections and recombination than coronaviruses that cause respiratory diseases. Different swine enteric coronaviruses can infect the same cells, providing the temporal and spatial conditions for genetic recombination [27]. These viruses also show tropism for the jejunum and ileum of piglets, providing favorable conditions for recombination during natural infections [4,28].

Because swine are raised and traded for meat globally, their infection by coronaviruses and their close interactions with humans pose a significant threat to public health, as exemplified by the outbreak of swine-origin human influenza virus in 2009 [29,30]. Despite this, no previous study has systematically examined the relationship between recombination and the coinfection modes of swine enteric coronaviruses, but have focused solely on recombinant strains of the same virus. Understanding the limitations of recombination at the level of coronavirus genera is essential for predicting their capacity to yield novel viruses. To address this knowledge gap, we collected intestinal and fecal samples from swine farms that had experienced severe diarrhea and piglet mortality. We analyzed the clinical diagnostic data and identified PEDV/PDCoV as a major coinfection. Using genomic, evolutionary, and recombination data, we identified important aspects of the major recombination mode of swine enteric coronaviruses and systematically analyzed the relationship between the recombination mode and the coinfection mode of swine enteric coronaviruses.

## Materials and methods

### Swine enteric coronavirus samples

Clinical samples were collected from pig farms in China in 2018–2021. All samples were tested for all three swine enteric coronaviruses, PEDV, TGEV and PDCoV. The coinfection rates were calculated as the ratio of the number of samples coinfected with two or more swine enteric coronaviruses to the number of samples infected with at least one swine enteric coronavirus. To avoid bias in our sample, we reviewed cases of co-infection with swine enteric coronaviruses reported in the literature and elsewhere.

### Sequence analysis

All 707 available PEDV sequences, 40 available TGEV sequences, 39 available SADS-CoV sequences, and 140 available PDCoV sequences were obtained from NCBI (https://www.ncbi.nlm.nih.gov) in August 2022 and used for a phylogenetic analysis and the classification of new lineages. All information (GenBank accession numbers; dates reported; countries; hosts) of swine enteric coronaviruses used for recombination analysis is given in Table S1, and the information of deltacoronaviruses used for recombination analysis is given in Table S2. All genomic sequences were aligned with MAFFT [31].

### Recombination analysis

Potential recombination within the whole-genome sequences was screened with seven methods: RDP, GENECONV, MaxChi, Bootscan, Chimera, SiScan and 3Seq implemented in the Recombination Detection Program version 4 (RDP4) [32]. We set *P* to < 0.01 to avoid inaccurate recombination events. The breakpoints were also defined with RDP4, and recombination events were deemed to have occurred when four of the seven methods reported recombination signals. Only recombination events with sufficient evidence (not partial or trace evidence in the same event) and for which both parental strains were unambiguously identified (i.e. no missing parental strain) were retained. To visualize the intraspecies recombination events and recombination hot spots, alignments of the full genomes were generated, and TBtools was used to show the areas of recombination [33]. Nucleotide sequence similarities were analyzed with Simplot v.3.5.1 (sliding window size, 500 bp; step size, 20 nucleotides; 1000 bootstrap replicates) [34], and the results were visualized with the GraphPad Prism software v.6.01.

### Phylogenetic analysis

Phylogenetic trees were constructed for the complete genomes of all swine enteric coronaviruses and deltacoronaviruses. ML phylogenetic trees were constructed with IQ-TREE, and the best-fitting nucleotide substitution model was determined automatically with the program after 1000 bootstrap replications [35]. The results were visualized with iTOL v.4 (http://itol.embl.de/) [36]. We used the same procedures to reconstruct an ML tree based on the deltacoronavirus sequences using the genome of IBV, a member of the genus *Gammacoronavirus*, as the outgroup.

### Visualization of genome models and recombination in swine enteric coronaviruses

The genome models of PEDV, TGEV, SADS-CoV, and PDCoV were determined with TBtools. Recombination events and breakpoint distributions were shown using ggplot2 and TBtools. The recombination frequency for each site was the percentage of recombinations breakpoint positions that occurred at a specific site from the total number of recombinants breakpoint positions. All the results were visualized with TBtools and the R programming language.

## Results

### Frequent coinfection and genetic diversity of swine enteric coronaviruses

**Figure 1.**
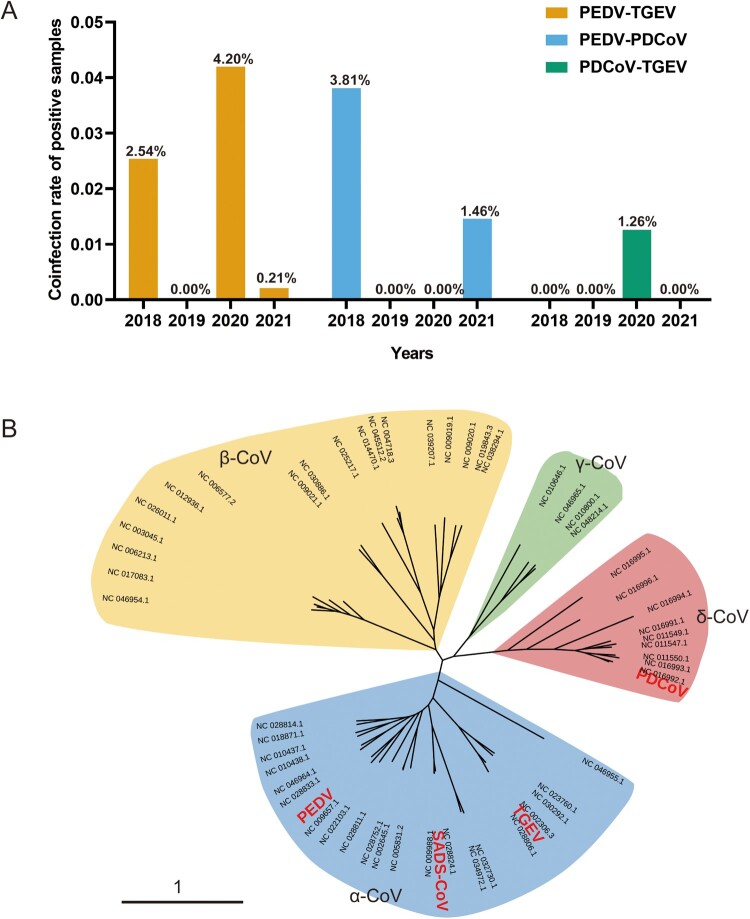
Coinfection analysis and phylogeny of swine enteric coronaviruses. (A) Coinfections of PEDV, PDCoV and TGEV detected in China in 2018–2021 in this study. (B) Phylogenetic tree of coronaviruses from different genera. Different colors represent different genera: *Alphacoronavirus* (α-CoV; blue), *Betacoronavirus*, (β-CoV; yellow), *Gammacoronavirus* (γ-CoV; green), and *Deltacoronavirus* (δ-CoV, red). PEDV, TGEV, SADS-CoV, and PDCoV are highlighted in red. The phylogenetic tree was visualized with iTOL v.4 (Interactive Tree of Life, http://itol.embl.de/).

We analyzed coinfections of swine enteric coronaviruses in 4,468 clinical samples collected between 2018 and 2021. The results revealed coinfection rates ranging from 0.21% to 4.20% in different years (Figure 1A), and the predominant mode of coinfection involved two swine enteric coronaviruses. The most common coinfection events occurred between PEDV and PDCoV (coinfection rate 1.50%), followed by PEDV and TGEV (coinfection rate 1.17%). These findings highlight the complexity of viral diarrhea pathogens on Chinese pig farms. Therefore, populations of diverse pathogens exist in the clinical setting, emphasizing the importance of monitoring this fluctuating infection spectrum to guide their clinical management.

To mitigate potential bias arising from testing a limited number of clinical samples within restricted sampling areas, we compared our results with recent surveys conducted in other countries and regions, including the United States [10,37], Mexico [38], South Korea [9], Vietnam[39], Thailand, the Philippines, Lao PDR [11], Japan [12], and mainland China [40–43]. The results showed that PEDV was the pathogen most frequently forming coinfections with PDCoV, and cases of multiple-virus infections involving PEDV and other viruses (such as TGEV) were also identified in these countries, with detection rates ranging from 0.12% to 18.80%. Our study did not include testing for SADS-CoV, a recently emerged coronavirus, because according to previous reports, its prevalence is limited [22]. Notably, in recent surveys, only three samples (0.1%) collected in Fujian Province, China, in 2017 showed dual infections of SADS-CoV and PEDV (Table 1).

**Table 1.** Findings from coinfections with swine enteric coronaviruses in previous studies.

Year	Country (region)	Pathogens	Positive Samples (coinfection rate)	Host	Renference
2011–2016	Vietnam	PDCoV/PEDV	11	Swine	[39]
2014	U.S.A	PDCoV/PEDV	19 (17%)	Swine	[37]
2014	U.S.A	PDCoV/PEDV	5 (19%)	Swine	[37]
2015	Thailand, Vietnam, Philippines and Lao PDR	PDCoV/PEDV	6 (17.14%)	Swine	[11]
2014–2016	South Korea	PDCoV/PEDV	43 (6.3%)	Swine	[9]
2014–2017	Mexico	PDCoV/PEDV	46 (5.2%)	Swine	[38]
		PDCoV/TGEV	9 (1.0%)	Swine	
		PDCoV/PEDV/TGEV	16 (1.8%)	Swine	
2014–2018	U.S.A	PDCoV/PEDV	307	Swine	[10]
2012–2015	China (Guangdong, Guangxi, Hainan)	PDCoV/PEDV	5 (1.28%)	Swine	[54]
2012–2018	China (Jiangxi, Zhejiang, Fujian, Guangdong, Hunan)	PDCoV/PEDV	380 (12.72%)	Swine	[40]
		PDCoV/TGEV	3 (0.1%)	Swine	
		PEDV/TGEV	9 (0.3%)	Swine	
		PEDV/SADS-CoV	3 (0.1%)	Swine	
		PEDV/PDCoV/TGEV	1 (0.03%)	Swine	
2015–2016	China (Shaanxi, Henan, Hubei)	PDCoV/PEDV	2 (2.9%)	Swine	[55]
2015–2018	China	PDCoV/PEDV	4.96%	Swine	[43]
2016–2017	China (Taiwan)	PDCoV/PEDV	14	Swine	[56]
2016–2017	China (Gansu, Qinghai, Sichuan)	PDCoV/PEDV	3 (1.59%)	Tibetan pigs	[57]
2016–2017	China (Heilongjiang, Liaoning, Beijing, Hebei, Henan, Shanxi, Shandong, Hubei, Anhui, Hunan, Jiangxi, Zhejiang, Jiangsu, Guangxi, Yunnan, Fujian, Sichuan, Gansu)	PDCoV/PEDV	34 (4.73%)	Swine	[41]

To gain a comprehensive understanding of the genetic diversity and evolutionary relationships among the swine enteric coronaviruses, we conducted an extensive analysis of 926 whole-genome sequences sourced from the National Center for Biotechnology Information (NCBI) database. A robust phylogenetic tree was constructed with IQ-TREE based on the full genomic sequences of these 926 coronaviruses (Figure S1). PEDV, TGEV, and SADS-CoV, which belong to the genus *Alphacoronavirus*, clustered with 229E, NL63, feline infectious peritonitis virus (FIPV), and certain bat-derived coronaviruses (Figure 1B), whereas OC43, HKU1, SARS-CoV, SARS-CoV-2, and MERS-CoV belong to the genus *Betacoronaviru*s. In contrast to the alphacoronaviruses and betacoronaviruses, the gammacoronaviruses primarily consist of avian coronaviruses (such as infectious bronchitis virus [IBV]) and coronaviruses isolated from aquatic animals, whales, and dolphins. The coronaviruses of wild-bird origin cluster among the deltacoronaviruses, which also include some swine-derived coronaviruses (Figure 1B).

Overall, alpha- and betacoronaviruses mainly include coronaviruses from mammals, whereas gamma- and deltacoronaviruses predominantly comprise avian-origin coronaviruses. Notably, all presently known swine enteric coronaviruses belong to either *Alphacoronavirus* or *Deltacoronavirus*, which also include coronaviruses of predominantly bat or bird origin. This observation underscores the genetic diversity and complexity of the swine enteric coronaviruses, highlighting their capacity for evolutionary change and adaptation to various host ranges.

### Recombination dynamics and frequencies in swine enteric coronaviruses

**Figure 2.**
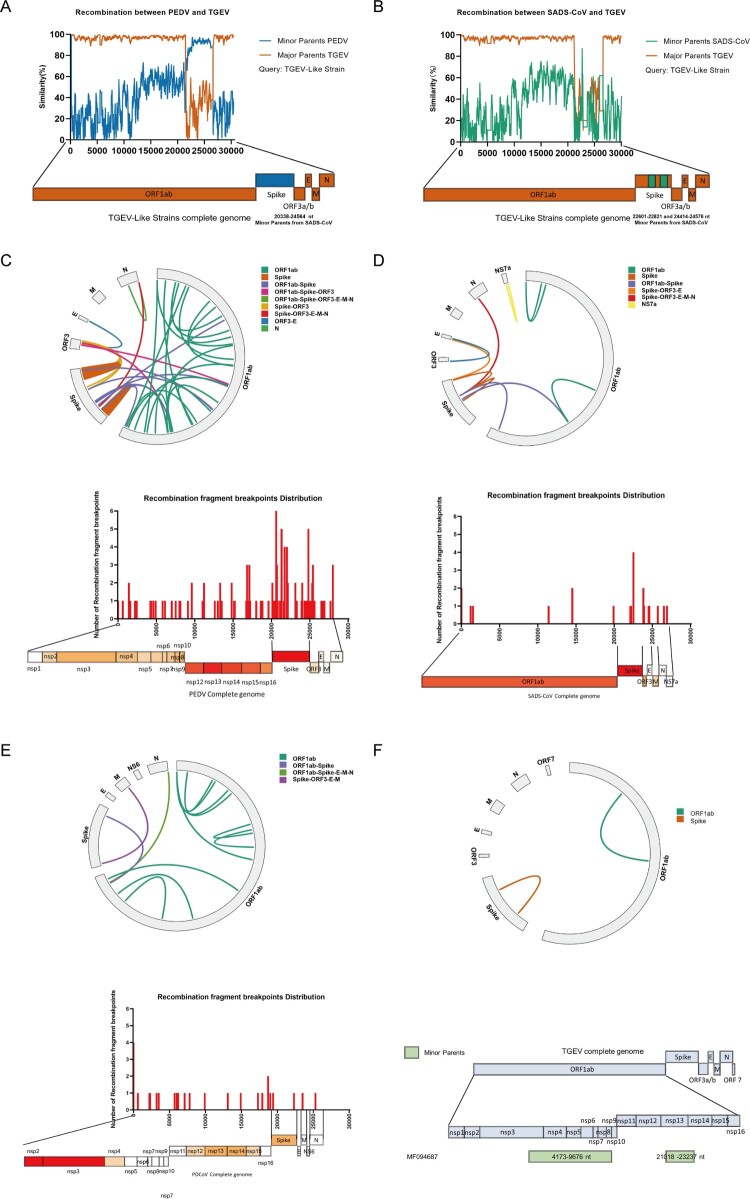
Comparison of interspecific and intraspecific recombinations of different swine enteric coronaviruses. Simplot analysis of possible interspecific recombination events in swine enteric coronaviruses. Differently colored lines represent different coronavirus species. (A) Major parent was TGEV, indicated in orange. Minor parent was PEDV, indicated in blue. (B) Major parent was TGEV, indicated in orange. Minor parent was SADS-CoV, indicated in green. (C) Intralineage recombinations of PEDV. (D) Intralineage recombinations of SADS-CoV. (E) Intralineage recombinations of PDCoV. (F) Intralineage recombinations of TGEV. Different breakpoints located in different genes are shown in different colors. Linkages represent recombination events, connecting the beginning and end positions of each event; histograms represent recombination breakpoints that occurred more than once; the genome model exhibits recombination hotspots, which are placed below the histograms. Color shades represent reorganization frequencies.

To understanding the dynamics of recombination among various swine enteric coronaviruses, we conducted an extensive analysis with RDP4, a sophisticated software designed specifically for the accurate detection and characterization of recombination. Two distinct recombination events were identified in the swine enteric coronaviruses, supported by robust statistical evidence from at least four tests with *P* values of < 0.01. The Simplot v3.5.1 program was used to validate these findings, and confirmed significant structural changes in two different swine enteric coronaviruses. Specifically, recombination was observed between PEDV and TGEV and between SADS-CoV and TGEV. An analysis of a TGEV-like strain (GenBank accession Number. MN692770) revealed a heterogeneous genome sequence resulting from recombination between a PEDV-related strain (GenBank accession number KY019624.1) and a TGEV-related strain (GenBank accession number KT696544). This recombination event led to the integration of two RNA fragments spanning the S gene, as shown in Figure 2A. The detailed information on recombination events is given in Table S3. Another TGEV-like strain displayed an integrated S gene sequence derived from SADS-CoV (GenBank accession number MF094685), indicating recombination between SADS-CoV and TGEV (Figure 2B and Table S3). Notably, the predominant recombination patterns observed in the swine enteric coronaviruses were between PEDV/TGEV and SADS-CoV/TGEV, with the breakpoints primarily located within the spike (S) gene region.

Previous studies have reported the occurrence of intraspecies recombination within the swine enteric coronaviruses [7]. In this study, we investigated intraspecies recombination patterns of different swine enteric coronaviruses by determining their recombination frequencies. Our findings identified notable differences in the recombination frequencies of these viruses. Specifically, we observed that the recombination frequency of TGEV was lower than those of the other swine enteric coronaviruses. Among the 40 TGEV strains analyzed, only two recombinant events were identified. In contrast, the recombination frequencies of PDCoV, PEDV, and SADS-CoV were 13/140, 54/707, and 11/39, respectively (Figure 2C–F). These results suggest that PDCoV and SADS-CoV, which cause emerging infectious diseases in swine, play significant roles in generating viral diversity through the formation of novel chimeric genomes via genetic recombination. Although numerous recombination events were detected in PEDV, possibly due to the abundance of PEDV strains, we consider that the recombination frequency in PEDV was relatively low, indicating that it has a more stable genome that is better adapted to its host than either PDCoV or SADS-CoV (Figure 2D,E).

Our findings also showed that the recombination breakpoints were scattered across the entire genomes of the swine enteric coronaviruses, with regions of notably frequent recombination within the S gene, an especially important protein of coronaviruses (Figure 2). These areas of high-frequency recombination varied among the different swine enteric coronaviruses. In PEDV, recombination “hot spots” were identified in the nsp12, nsp13, and nsp14 regions, which are crucial for PEDV replication. Conversely, the primary recombination hot spots were situated in the nsp2 and nsp3 regions of PDCoV (Figure 2C,E).

### Recombination induces host range expansion and viral evolution in deltacoronaviruses

**Figure 3.**
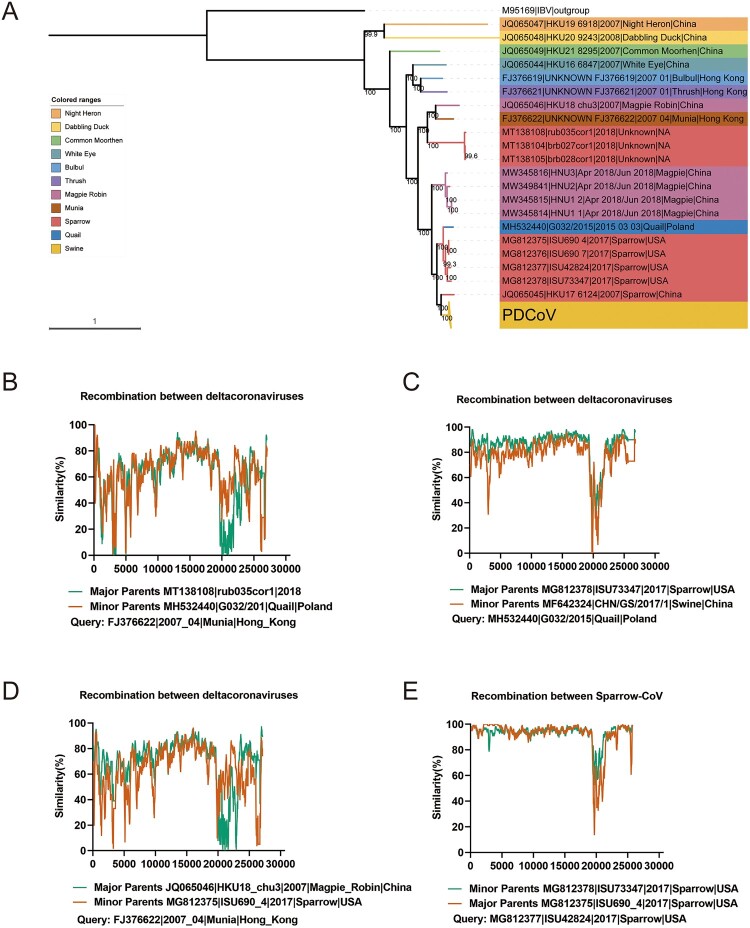
Phylogenetic tree and interspecific recombination of known deltacoronaviruses. (A) Maximum likelihood phylogenetic tree of deltacoronavirus genomes. The best-fitting nucleotide substitution model was determined automatically by the program after 1000 bootstrap replications, and the phylogenetic tree was visualized with iTOL v.4 (Interactive Tree of Life, http://itol.embl.de/). Numbers at the branches represent bootstra*p* values obtained in the phylogenetic analysis. Different colors represent different deltacoronavirus species. The PDCoVs collapsed into one node are shown with a yellow triangle, and sparrow coronavirus is shown in pink. (B–E) Simplot analysis of possible inter- and intraspecific recombination events in deltacoronaviruses. All major parents of the recombinations are indicated in green (GenBank accession numbers JQ065046, MG812378, MT138108, and MG812375). All minor parents of the recombinations are indicated in orange (GenBank accession numbers MG812375, MF642324, MH532440, and MG812378).

Our results clarified the recombination patterns involving the S genes of PEDV and TGEV and those of SADS-CoV and TGEV. These patterns underscore a clear inclination for interspecies recombination within the same genus of swine enteric coronavirus, whereas recombination events across different genera are less frequent. To delve deeper into this phenomenon, we subjected the entire deltacoronaviruses to phylogenetic and recombination analyses. To comprehensively assess the genetic diversity of the deltacoronaviruses across diverse hosts, we constructed a phylogenetic tree from 196 complete deltacoronavirus genomes, using the IBV genome (a member of the genus *Gammacoronavirus*) as the outgroup. We used the maximum-likelihood (ML) methodology in IQ-TREE to phylogenetically analyze the complete deltacoronavirus genomes, and identified specific patterns (Figure 3A). Based on the topologies of the phylogenetic tree and considering the viral hosts, we classified the deltacoronaviruses into two distinct groups: PDCoVs and bird coronaviruses. Interestingly, the sparrow coronavirus strains showed inconsistent topologies on the phylogenetic trees, with outlier sequences observed within other bird coronavirus subgroups. This inconsistency may have arisen from cross-species transmission events or genomic recombination events (Figure 3A).

To clarify the interspecific recombination patterns across different deltacoronaviruses, we constructed a multiple sequence alignment and used RDP4 to identify independent recombination events. Our analysis revealed four distinct recombination events in various deltacoronaviruses, each supported by robust statistical evidence from at least four tests, with a significance threshold (*P* value) of < 0.01 for each test. Notably, two of these events indicated the existence of a potential long recombination segment spanning aligned positions 20,000–25000, predominantly located within the S gene of munia coronavirus (GenBank accession number FJ396622). This recombinant segment probably originated from sparrow coronavirus (GenBank accession number MG812375) or quail coronavirus (GenBank accession number MH532440) (Figure 3B,D). Because the receptor-binding domain of the virus occurs in the S protein, the emergence of recombined sequences in this region may induce changes in receptor binding, potentially facilitating cross-species transmission. Surprisingly, we also identified a short potential recombination segment spanning aligned positions 21,000–22,000 of quail coronavirus, with sparrow coronavirus and PDCoV (GenBank accession number MF642324) as the putative recombinant parents (Figure 3C). We also detected additional potential recombination events within the sparrow coronaviruses, suggesting that the genomic stability of sparrow coronaviruses is lower than normal levels, reliance on recombination to enhance adaptability to the host (Figure 3E). The detailed information on recombination events is given in Table S4. Our findings underscore the frequent incidence of recombination events among deltacoronaviruses, particularly within the S gene region responsible for encoding the receptor-binding protein. These recombination events potentially played a role in the emergence of novel viruses capable of infecting new hosts. Avian species, particularly sparrows, often act as crucial transmission vectors and reservoirs for viral infections, owing to their gregarious behavior and capacity for long-distance travel.

### Human PDCoVs have undergone interlineage recombination during their evolutionary history

**Figure 4.**
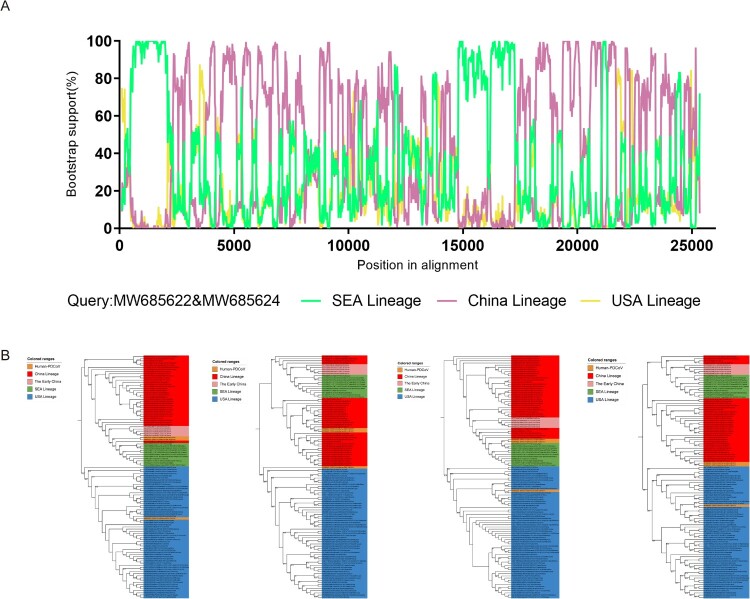
Recombinant features of human PDCoVs. (A) Structure of the PDCoV genome and bootscanning recombination analysis based on genomic sites. Colored broken lines represent different lineages: green indicates the SEA lineage, purple indicates the China lineage, and yellow indicates the USA lineage. (B) ML phylogenetic trees were inferred for the different recombinant regions: nucleotides 1–2320, 2320–14780, 14780–17280, and 17280 to the end. The SEA lineage is indicated in green, the China lineage in red, the USA lineage in blue, and the early China lineage in pink. Orange indicates three human PDCoV strains.

Lednicky et al. recently reported the detection of PDCoV in plasma samples isolated from three Haitian children presenting with acute undifferentiated febrile illness, indicating that human can be infected by PDCoV [44]. Recombination is a mechanism that contributes to viral genetic diversity and facilitates viral adaptation to novel hosts [19]. To assess the potential influence of recombination on the evolutionary dynamics of human PDCoV, we employed SimPlot v3.5.1 to conduct a comprehensive comparison of similarities across the viral genomes. This analysis revealed a mosaic structure suggestive of recombination events in two human PDCoV isolates (GenBank accession numbers MW685622 and MW685624). Notably, these two viruses showed identical recombination patterns, strongly indicating a single recombination event. The precise recombination breakpoints were identified at nucleotide positions 2320, 14780, and 17280, located within the genomes’ nsp3 and nsp14/nsp15 regions (Figure 4A). A phylogenetic analysis of the corresponding parental regions provided additional support for the occurrence of this recombination event. Intriguingly, this recombination event involved two distinct lineages of PDCoV, specifically the China lineage and the SEA lineage, suggesting that recombination events between different PDCoV strains may contribute to the expansion of the viral host range (Figure 4B).

Conversely, no evidence of recombination was observed in another human PDCoV strain (GenBank accession number MW685623) analyzed with the same methodology. This observation suggests that different human PDCoV strains have diverse evolutionary origins, and that mutations may also play a role in viral adaptation to the host. Notably, an amino acid alignment analysis identified two unique mutations (H139R in nsp2 and V922A in nsp3), as well as four amino acid mutations (I308 T and Y317D in nsp3, V30A in nsp15, and L38P in S), in human PDCoVs relative to most other PDCoVs, with the exception of the Chinese strain KY065120 (Table 2).

**Table 2.** Divergent amino acid mutations in human PDCoV compared to other lineages of PDCoVs.

Position	nsp2	nsp3			nsp15	S	
	139	308	317	922	30	38	551
most of China lineage	R/C	T/I (2)	D/Y	A	A/V (2)	L/P	A/V
Early China lineage	R	T	D	A	A	P	A
USA lineage	R	T/I	D	A	A	P	V
SEA lineage	C/R	T	D	A	A	P	A/V
MW685624&MW685622 (Human PDCoV)	H	I	Y	V	V	L	A
KY065120	H	I	Y	V	V	L	A
MW685622 (Human PDCoV)	H	I	Y	V	V	L	A
KR150443	R	T	D	A	A	P	V

## Discussion

Swine diarrheal disease poses a severe threat to the pig farming industry, with swine enteric coronaviruses emerging as the primary causative agents, particularly affecting piglets and newborns [45]. Notable pathogens include PEDV, TGEV, PDCoV, and SADS-CoV. Infections of these coronaviruses produce clinical symptoms such as diarrhea, vomiting, dehydration, and weight loss, with mortality rates up to 100% [40,41]. Our study underscores the frequent occurrence of coinfections involving the swine enteric coronaviruses, with dual infections the predominant form. The most frequent combinations involve PEDV and PDCoV, followed by PDCoV and TGEV, highlighting the intricate nature of viral diarrhea pathogens within pig farms. Although sampling bias, like most other epidemiological studies, cannot be completely avoided, we have tried our best to minimize it. For example, to demonstrate the pattern of swine enteric coronaviruses coinfection that we detected, we compared our results with recent surveys conducted in other countries and regions to minimize the effects of sampling bias. Our findings, cross-referenced with those of global studies, reveal similar coinfection patterns, especially involving PEDV and other viruses, such as PDCoV, indicating that coinfection events are not limited to specific geographic regions but are a global phenomenon. The consistent identification of these coinfection patterns emphasizes the urgent need for comprehensive surveillance and effective control measures to address the complex dynamics of the swine enteric coronaviruses. It is noteworthy that coinfections can contribute to the evolution of initially nonpathogenic enteric viruses into highly virulent and pathogenic strains. A prime example is the emergence of PDCoV in China and Hong Kong, where the virus circulated in pig herds for several years without causing observable clinical symptoms [46]. However, severe outbreaks of PDCoV have occurred in various different states of the USA since 2014 [47]. This change in virulence is attributable to previous exposure to PEDV or other coinfecting swine enteric viruses. Hence, coinfections involving swine enteric coronaviruses present to the prevention and control of swine diarrhea.

Recombination is a fundamental process in the evolution of RNA viruses, and plays a crucial role in shaping their genetic diversity and facilitating their adaptation [48]. Coinfection by multiple viruses, typically different swine enteric coronaviruses infecting the same tissues and cells in vivo, provides ample opportunities for recombination events [2,49]. Recombination can occur both within and between different viral subgroups or species, leading to the emergence of interspecies and intraspecies recombination patterns. The process of coinfection, particularly with different coronaviruses, facilitates recombination events during viral replication. These generate multiple subgenomic RNAs that are highly susceptible to recombinant exchange with the corresponding genes of other coronaviruses, primarily through template switching mechanisms [50]. A notable emergence of novel swine enteric coronaviruses was observed in central Eastern European countries between 2012 and 2016, resulting from recombination events involving PEDV and TGEV. These recombinant viruses spread extensively within the region [51]. In the present study, we identified distinct recombination patterns between different swine enteric coronaviruses, specifically involving PEDV and TGEV, as well as SADS-CoV and TGEV. Surprisingly, these recombination events primarily occurred within the S gene region and deviated from the observed coinfection patterns. Based on our findings, we hypothesize that interspecific recombination is more likely to occur between different viral species within the same genus, whereas recombination between swine enteric coronaviruses from different genera is less likely. This observation suggests that the genetic compatibility required for recombination may be influenced by the taxonomic relationships of the viruses involved.

Another intriguing observation of our study was the higher intraspecific recombination frequencies shown by PDCoV and SADS-CoV than by TGEV and PEDV. This finding suggests a higher propensity for genetic exchange in these two coronaviruses, implying greater potential for the generation of diverse viral variants through recombination events. Specifically, our study identified specific regions within key viral components in which recombination events occur at higher frequencies: the nonstructural proteins (nsp12, nsp13, and nsp14 regions in PEDV; nsp2 and nsp3 in PDCoV) and the S gene. Notably, we identified two human PDCoVs with a recombination origin involving breakpoints in nsp3 and nsp14/nsp15, which play important roles in viral replication, gene expression, and in triggering the innate immune response [52]. The susceptibility of nonstructural proteins to recombination may be attributable to their essential role in viral replication processes [53]. The intricate interplay between these proteins facilitates genetic exchange and promotes the emergence of novel variants. The S protein, which is responsible for viral entry and immune recognition, displays genetic variations that play crucial roles in viral adaptation and the evasion of the host immune responses. Our study also presents compelling evidence for interspecies recombination within the deltacoronaviruses, and provides novel insights into the occurrence of recombination events between different coronavirus species within the same genus. Notably, our study shows hitherto unreported instances of interspecies recombination involving CoV strains from the magpie robin and sparrow, sparrow and swine, and sparrow and quail. These findings extend our understanding of the recombination landscape within the deltacoronaviruses. However, we acknowledge that further sequencing efforts and comprehensive analyses of recombination patterns are required to validate and consolidate our observations. Our results also suggest a potential association between the active recombination of the deltacoronaviruses and avian species, particularly sparrows, which share ecological niches with domestic mammals. These findings suggest the involvement of birds as especial hosts for recombination events, acting as reservoirs for genetic exchange between coronaviruses of different species. In light of the virus–host ecology of the deltacoronaviruses, it is imperative to strengthen proactive surveillance efforts and to monitor the emergence of viral variants, interspecies transmission dynamics, and the potential for pandemic risks in animals, including avian populations.

**Figure 5.**
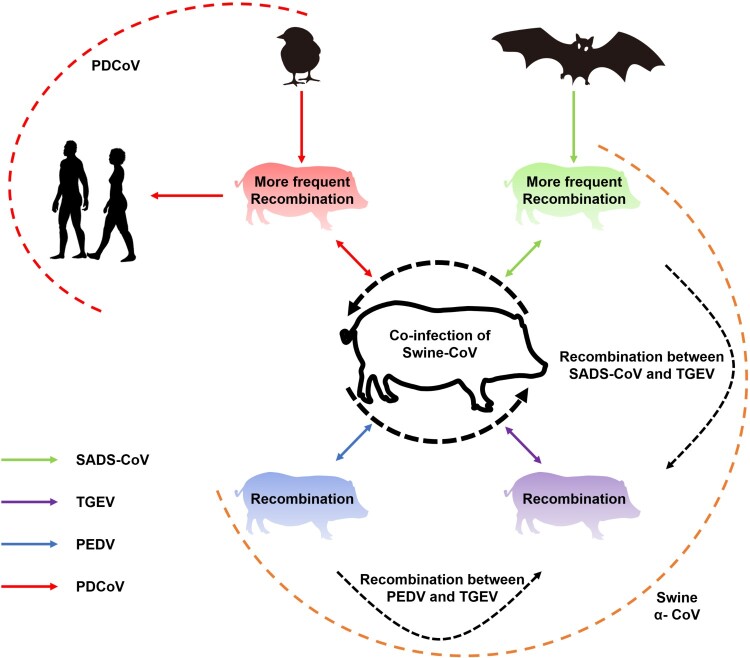
Intra- and interspecies transmission and recombination of different swine enteric coronaviruses. Green, purple, blue, and red arrows represent the transmission of SADS-CoV, TGEV, PEDV, and PDCoV, respectively, between bats, sparrows, and swine (shown in the legend on the side of the figure). Unbroken arrows represent confirmed transmission between the two species in question, and broken arrows represent suspected interspecies recombination.

Given the cocirculation and coinfection of multiple species of coronaviruses in diverse animal populations, which frequently interact with each other in the wild, it seems only a matter of time before the emergence of the next recombinant coronavirus triggers another human epidemic outbreak. As shown in Figure 5, swine enteric coronaviruses provide a valuable model in which to investigate the intricate relationships between viral coinfections and recombination dynamics. The recurrent recombination events observed in swine enteric coronaviruses have led to the emergence of highly diverse novel viruses displaying unpredictable changes in virulence. Consequently, a comprehensive re-evaluation of global strategies for the prevention and control of swine enteric coronavirus is warranted. The establishment of a robust surveillance network is also crucial for the detection and prediction of the emergence of potentially highly virulent recombinant coronaviruses originating from animal reservoirs, to mitigate the risk of future pandemics in humans.

## Supplementary Material

Supplementary_materials

## Data Availability

The data that support for the findings of this study are all contained in the manuscript.
