# Supplementary material for: Coinfection and nonrandom recombination drive the evolution of swine enteric coronaviruses
Source: Emerg Microbes Infect. 2024 Mar 22;13(1):2332653. doi: 10.1080/22221751.2024.2332653 (PMC10977008; doi:10.1080/22221751.2024.2332653)
Supplement: Supplementary_materials [file TEMI_A_2332653_SM5715.doc]

**Supplementary materials**

**Figure S1.** Phylogenetic tree of swine enteric coronaviruses.

**Table S1.** Information of swine enteric coronaviruses used in this study.

**Table S2.** Information of all deltacoronaviruses used in this study.

**Table S3.** Information of recombination events detected in swine enteric coronaviruses using RDP4.

**Table S4.** Information of recombination events detected in deltacoronaviruses using RDP4.

**Figure S1**

**
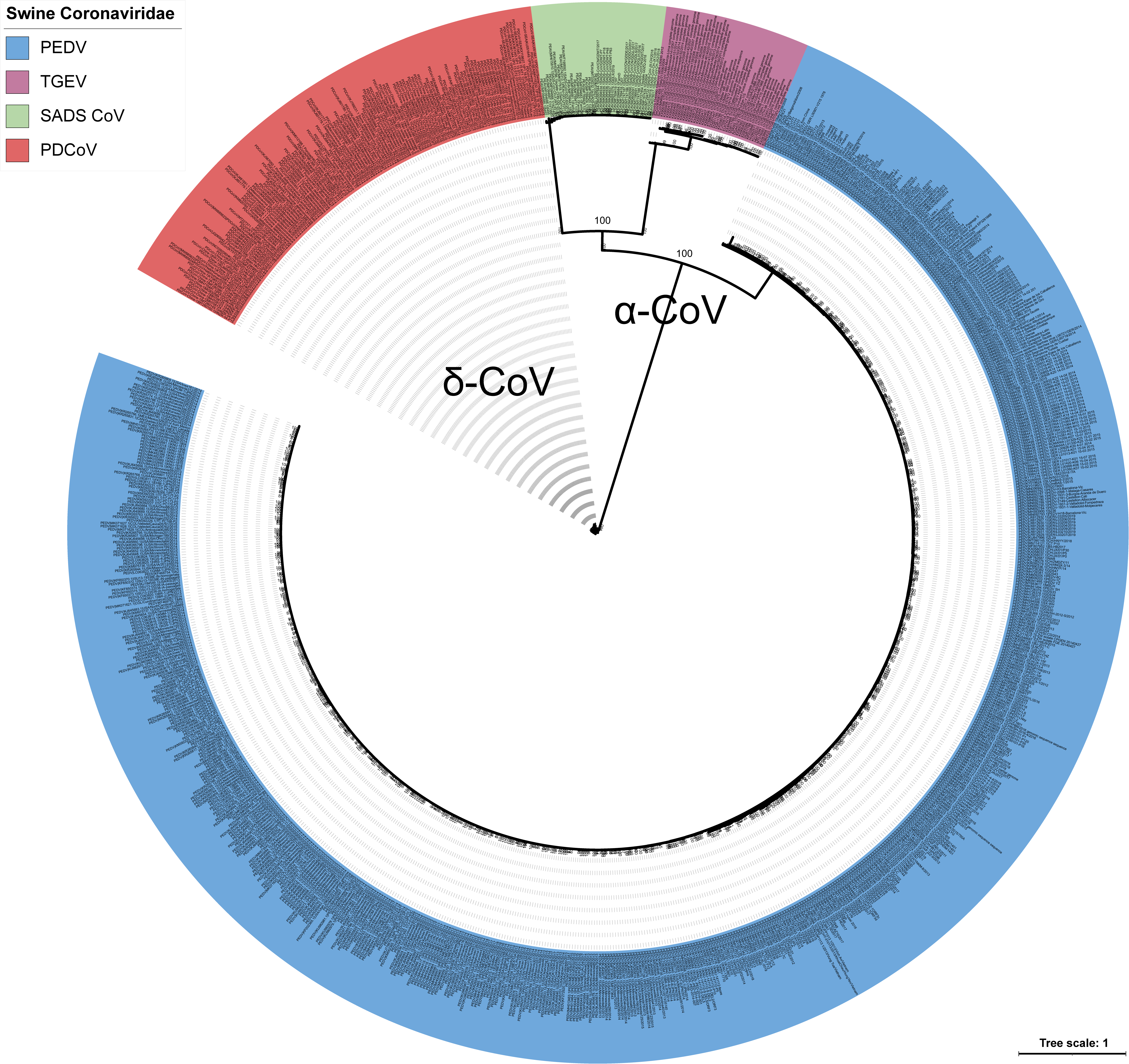
**

**Figure S1.** **Phylogenetic tree of swine enteric coronaviruses.** Different colors represent different coronavirus species, PEDV (blue), TGEV (Purple), SADS-CoV (green), and PDCoV (Pink), and the phylogenetic trees were visualized using iTOL v.4 (Interactive Tree of Life, <http://itol.embl.de/>). Numbers at the branches represent bootstrap values obtained in the phylogenetic analysis. All available swine enteric coronaviruses genome sequences up to August 2022 were retrieved from NCBI GenBank (https://www.ncbi.nlm.nih.gov/). A total of 707 available PEDV sequences，40 available TGEV sequences，39 available SADS-CoV sequences, and 140 available PDCoV sequences on GenBank.

**Table S1.** **Information of** **swine enteric coronaviruses used in this study.**

| **GenBank ID** | **Reported time** | **Country/Region** | **Host** | **Organism** |
| --- | --- | --- | --- | --- |
| **DQ201447.1** | **N/A** | **N/A** | **Swine** | **Transmissible gastroenteritis virus** |
| **DQ443743.1** | **N/A** | **China** | **Swine** | **Transmissible gastroenteritis virus** |
| **DQ811785.1** | **N/A** | **USA** | **Swine** | **TGEV Miller M6** |
| **DQ811786.2** | **1987** | **USA** | **Swine** | **TGEV Miller M60** |
| **DQ811788.1** | **N/A** | **N/A** | **Swine** | **TGEV Purdue P115** |
| **DQ811789.2** | **1952** | **USA** | **Swine** | **TGEV virulent Purdue** |
| **EF185992.1** | **N/A** | **China** | **Swine** | **Porcine epidemic diarrhea virus** |
| **EU074218.2** | **N/A** | **China** | **Swine** | **Transmissible gastroenteritis virus** |
| **FJ755618.2** | **1973** | **China** | **Swine** | **Transmissible gastroenteritis virus** |
| **GU937797.1** | **N/A** | **N/A** | **Swine** | **Porcine epidemic diarrhea virus** |
| **HM776941.1** | **2009** | **China** | **Swine** | **Transmissible gastroenteritis virus** |
| **HQ462571.1** | **N/A** | **China** | **Swine** | **Transmissible gastroenteritis virus** |
| **JN547228.1** | **1986** | **China** | **Swine** | **Porcine epidemic diarrhea virus** |
| **JN825712.1** | **2011/4/25** | **China** | **Swine** | **Porcine epidemic diarrhea virus** |
| **JQ065042.2** | **2009** | **Hong Kong** | **Swine** | **Porcine coronavirus HKU15** |
| **JQ282909.1** | **2011/5/3** | **China** | **Swine** | **Porcine epidemic diarrhea virus** |
| **JX088695.1** | **2012** | **China** | **Swine** | **Porcine epidemic diarrhea virus** |
| **JX112709.1** | **2012/2/11** | **China** | **Swine** | **Porcine epidemic diarrhea virus** |
| **JX188454.1** | **2011/5/1** | **China** | **Swine** | **Porcine epidemic diarrhea virus** |
| **JX261936.1** | **2011/2/10** | **China** | **Swine** | **Porcine epidemic diarrhea virus** |
| **JX489155.1** | **2011/2/1** | **China** | **Swine** | **Porcine epidemic diarrhea virus** |
| **JX524137.1** | **2011/4/10** | **China** | **Swine** | **Porcine epidemic diarrhea virus** |
| **JX560761.1** | **2012/2/1** | **China** | **Swine** | **Porcine epidemic diarrhea virus** |
| **KC109141.1** | **N/A** | **China** | **Swine** | **Porcine epidemic diarrhea virus** |
| **KC140102.1** | **2012/8/20** | **China** | **Swine** | **Porcine epidemic diarrhea virus** |
| **KC189944.1** | **N/A** | **N/A** | **Swine** | **Porcine epidemic diarrhea virus** |
| **KC196276.1** | **2011/5/1** | **China** | **Swine** | **Porcine epidemic diarrhea virus** |
| **KC210145.1** | **2012/3/15** | **China** | **Swine** | **Porcine epidemic diarrhea virus** |
| **KC210146.1** | **2008** | **China** | **Swine** | **Porcine epidemic diarrhea virus** |
| **KC210147.1** | **2012** | **China** | **Swine** | **Porcine epidemic diarrhea virus** |
| **KC962433.1** | **2012/5/5** | **China** | **Swine** | **Transmissible gastroenteritis virus** |
| **KF267450.1** | **2013/5/29** | **USA** | **Swine** | **Porcine epidemic diarrhea virus** |
| **KF272920.1** | **2013/5/10** | **USA** | **Swine** | **Porcine epidemic diarrhea virus** |
| **KF384500.1** | **2012/3/1** | **China** | **Swine** | **Porcine epidemic diarrhea virus** |
| **KF468752.1** | **2013/6/19** | **USA** | **Swine** | **Porcine epidemic diarrhea virus** |
| **KF468753.1** | **2013** | **USA** | **Swine** | **Porcine epidemic diarrhea virus** |
| **KF468754.1** | **2013** | **USA** | **Swine** | **Porcine epidemic diarrhea virus** |
| **KF650370.1** | **2013/5/16** | **USA** | **Swine** | **Porcine epidemic diarrhea virus** |
| **KF650372.1** | **N/A** | **USA** | **Swine** | **Porcine epidemic diarrhea virus** |
| **KF650373.1** | **2013/6/6** | **USA** | **Swine** | **Porcine epidemic diarrhea virus** |
| **KF650374.1** | **N/A** | **USA** | **Swine** | **Porcine epidemic diarrhea virus** |
| **KF650375.1** | **N/A** | **USA** | **Swine** | **Porcine epidemic diarrhea virus** |
| **KF760557.2** | **2013/3/1** | **China** | **Swine** | **Porcine epidemic diarrhea virus** |
| **KF761675.1** | **2013/9/20** | **China** | **Swine** | **Porcine epidemic diarrhea virus** |
| **KF840537.1** | **2012/3/4** | **China** | **Swine** | **Porcine epidemic diarrhea virus** |
| **KJ020932.1** | **2013/3/1** | **China** | **Swine** | **Porcine epidemic diarrhea virus** |
| **KJ158152.1** | **2011/10/20** | **China** | **Swine** | **Porcine epidemic diarrhea virus** |
| **KJ184549.1** | **2013** | **USA** | **Swine** | **Porcine epidemic diarrhea virus** |
| **KJ196348.1** | **2013/2/5** | **China** | **Swine** | **Porcine epidemic diarrhea virus** |
| **KJ399978.1** | **2014/1/15** | **USA** | **Swine** | **Porcine epidemic diarrhea virus** |
| **KJ408801.1** | **2014/1/23** | **USA** | **Swine** | **Porcine epidemic diarrhea virus** |
| **KJ462462.1** | **2014/1/31** | **USA** | **Swine** | **Porcine coronavirus HKU15** |
| **KJ481931.1** | **2014/1/4** | **USA** | **Swine** | **Porcine coronavirus HKU15** |
| **KJ567050.1** | **2014/2/20** | **USA** | **Swine** | **Porcine coronavirus HKU15** |
| **KJ569769.1** | **2014/2/13** | **USA** | **Swine** | **Porcine coronavirus HKU15** |
| **KJ584355.1** | **2014/2/12** | **USA** | **Swine** | **Porcine coronavirus HKU15** |
| **KJ584356.1** | **2014/2/20** | **USA** | **Swine** | **Porcine coronavirus HKU15** |
| **KJ584357.1** | **2014/3/7** | **USA** | **Swine** | **Porcine coronavirus HKU15** |
| **KJ584358.1** | **2014/2/18** | **USA** | **Swine** | **Porcine coronavirus HKU15** |
| **KJ584359.1** | **2014/2/21** | **USA** | **Swine** | **Porcine coronavirus HKU15** |
| **KJ584361.1** | **2013/6/14** | **USA** | **Swine** | **Porcine epidemic diarrhea virus** |
| **KJ588062.1** | **2014/2/1** | **South Korea** | **Swine** | **Porcine epidemic diarrhea virus** |
| **KJ588063.1** | **2014/2/1** | **South Korea** | **Swine** | **Porcine epidemic diarrhea virus** |
| **KJ588064.1** | **2014/2/1** | **South Korea** | **Swine** | **Porcine epidemic diarrhea virus** |
| **KJ601777.1** | **2014/1/8** | **USA** | **Swine** | **Porcine coronavirus HKU15** |
| **KJ601778.1** | **2014/1/8** | **USA** | **Swine** | **Porcine coronavirus HKU15** |
| **KJ601779.1** | **2014/1/11** | **USA** | **Swine** | **Porcine coronavirus HKU15** |
| **KJ601780.1** | **2014/1/26** | **USA** | **Swine** | **Porcine coronavirus HKU15** |
| **KJ620016.1** | **2014/3/18** | **USA** | **Swine** | **Porcine coronavirus HKU15** |
| **KJ623926.1** | **2014** | **South Korea** | **Swine** | **Porcine epidemic diarrhea virus** |
| **KJ645635.1** | **2013/6/8** | **USA** | **Swine** | **Porcine epidemic diarrhea virus** |
| **KJ645636.1** | **2013/7/14** | **USA** | **Swine** | **Porcine epidemic diarrhea virus** |
| **KJ645637.1** | **2013/7/15** | **USA** | **Swine** | **Porcine epidemic diarrhea virus** |
| **KJ645638.1** | **2013/7/22** | **USA** | **Swine** | **Porcine epidemic diarrhea virus** |
| **KJ645639.1** | **2013/7/22** | **USA** | **Swine** | **Porcine epidemic diarrhea virus** |
| **KJ645640.1** | **2013/7/24** | **USA** | **Swine** | **Porcine epidemic diarrhea virus** |
| **KJ645641.1** | **2013/8/21** | **USA** | **Swine** | **Porcine epidemic diarrhea virus** |
| **KJ645642.1** | **2013/8/26** | **USA** | **Swine** | **Porcine epidemic diarrhea virus** |
| **KJ645643.1** | **2013/9/1** | **USA** | **Swine** | **Porcine epidemic diarrhea virus** |
| **KJ645644.1** | **2013/9/7** | **USA** | **Swine** | **Porcine epidemic diarrhea virus** |
| **KJ645645.1** | **2013/9/28** | **USA** | **Swine** | **Porcine epidemic diarrhea virus** |
| **KJ645646.1** | **2013/10/5** | USA | **Swine** | **Porcine epidemic diarrhea virus** |
| **KJ645647.1** | **2013/10/14** | **USA** | **Swine** | **Porcine epidemic diarrhea virus** |
| **KJ645648.1** | **2013/10/20** | **USA** | **Swine** | **Porcine epidemic diarrhea virus** |
| **KJ645649.1** | **2013/10/20** | **USA** | **Swine** | **Porcine epidemic diarrhea virus** |
| **KJ645650.1** | **2013/10/23** | **USA** | **Swine** | **Porcine epidemic diarrhea virus** |
| **KJ645651.1** | **2013/10/23** | **USA** | **Swine** | **Porcine epidemic diarrhea virus** |
| **KJ645652.1** | **2013/11/2** | **USA** | **Swine** | **Porcine epidemic diarrhea virus** |
| **KJ645653.1** | **2013/11/3** | **USA** | **Swine** | **Porcine epidemic diarrhea virus** |
| **KJ645654.1** | **2013/11/4** | **USA** | **Swine** | **Porcine epidemic diarrhea virus** |
| **KJ645655.1** | **2013/11/5** | **USA** | **Swine** | **Porcine epidemic diarrhea virus** |
| **KJ645656.1** | **2013/11/11** | **USA** | **Swine** | **Porcine epidemic diarrhea virus** |
| **KJ645657.1** | **2013/11/12** | **USA** | **Swine** | **Porcine epidemic diarrhea virus** |
| **KJ645658.1** | **2013/11/18** | **USA** | **Swine** | **Porcine epidemic diarrhea virus** |
| **KJ645659.1** | **2013/11/19** | **USA** | **Swine** | **Porcine epidemic diarrhea virus** |
| **KJ645660.1** | **2013/11/19** | **USA** | **Swine** | **Porcine epidemic diarrhea virus** |
| **KJ645661.1** | **2013/11/20** | **USA** | **Swine** | **Porcine epidemic diarrhea virus** |
| **KJ645662.1** | **2013/11/23** | **USA** | **Swine** | **Porcine epidemic diarrhea virus** |
| **KJ645663.1** | **2013/11/23** | **USA** | **Swine** | **Porcine epidemic diarrhea virus** |
| **KJ645664.1** | **2013/11/24** | **USA** | **Swine** | **Porcine epidemic diarrhea virus** |
| **KJ645665.1** | **2013/11/24** | **USA** | **Swine** | **Porcine epidemic diarrhea virus** |
| **KJ645666.1** | **2013/11/24** | **USA** | **Swine** | **Porcine epidemic diarrhea virus** |
| **KJ645667.1** | **2013/11/28** | **USA** | **Swine** | **Porcine epidemic diarrhea virus** |
| **KJ645668.1** | **2013/11/25** | **USA** | **Swine** | **Porcine epidemic diarrhea virus** |
| **KJ645669.1** | **2013/11/27** | **USA** | **Swine** | **Porcine epidemic diarrhea virus** |
| **KJ645670.1** | **2013/11/27** | **USA** | **Swine** | **Porcine epidemic diarrhea virus** |
| **KJ645671.1** | **2013/11/30** | **USA** | **Swine** | **Porcine epidemic diarrhea virus** |
| **KJ645672.1** | **2013/11/28** | **USA** | **Swine** | **Porcine epidemic diarrhea virus** |
| **KJ645673.1** | **2013/11/28** | **USA** | **Swine** | **Porcine epidemic diarrhea virus** |
| **KJ645674.1** | **2013/11/30** | **USA** | **Swine** | **Porcine epidemic diarrhea virus** |
| **KJ645675.1** | **2013/12/3** | **USA** | **Swine** | **Porcine epidemic diarrhea virus** |
| **KJ645676.1** | **2013/12/2** | **USA** | **Swine** | **Porcine epidemic diarrhea virus** |
| **KJ645677.1** | **2013/12/2** | **USA** | **Swine** | **Porcine epidemic diarrhea virus** |
| **KJ645678.1** | **2013/12/4** | **USA** | **Swine** | **Porcine epidemic diarrhea virus** |
| **KJ645679.1** | **2013/12/7** | **USA** | **Swine** | **Porcine epidemic diarrhea virus** |
| **KJ645680.1** | **2013/12/4** | **USA** | **Swine** | **Porcine epidemic diarrhea virus** |
| **KJ645681.1** | **2013/12/4** | **USA** | **Swine** | **Porcine epidemic diarrhea virus** |
| **KJ645682.1** | **2013/12/4** | **USA** | **Swine** | **Porcine epidemic diarrhea virus** |
| **KJ645683.1** | **2013/12/8** | **USA** | **Swine** | **Porcine epidemic diarrhea virus** |
| **KJ645684.1** | **2013/12/11** | **USA** | **Swine** | **Porcine epidemic diarrhea virus** |
| **KJ645685.1** | **2013/12/11** | **USA** | **Swine** | **Porcine epidemic diarrhea virus** |
| **KJ645686.1** | **2013/12/10** | **USA** | **Swine** | **Porcine epidemic diarrhea virus** |
| **KJ645687.1** | **2013/12/10** | **USA** | **Swine** | **Porcine epidemic diarrhea virus** |
| **KJ645688.1** | **2013/12/11** | **USA** | **Swine** | **Porcine epidemic diarrhea virus** |
| **KJ645689.1** | **2013/12/11** | **USA** | **Swine** | **Porcine epidemic diarrhea virus** |
| **KJ645690.1** | **2013/12/11** | **USA** | **Swine** | **Porcine epidemic diarrhea virus** |
| **KJ645691.1** | **2013/12/14** | **USA** | **Swine** | **Porcine epidemic diarrhea virus** |
| **KJ645692.1** | **2013/12/15** | **USA** | **Swine** | **Porcine epidemic diarrhea virus** |
| **KJ645693.1** | **2013/12/15** | **USA** | **Swine** | **Porcine epidemic diarrhea virus** |
| **KJ645694.1** | **2013/12/15** | **USA** | **Swine** | **Porcine epidemic diarrhea virus** |
| **KJ645695.1** | **2013/12/29** | **USA** | **Swine** | **Porcine epidemic diarrhea virus** |
| **KJ645696.1** | **2013/12/29** | **USA** | **Swine** | **Porcine epidemic diarrhea virus** |
| **KJ645697.1** | **2013/11/12** | **USA** | **Swine** | **Porcine epidemic diarrhea virus** |
| **KJ645698.1** | **2014/1/1** | **USA** | **Swine** | **Porcine epidemic diarrhea virus** |
| **KJ645699.1** | **2013/12/15** | **USA** | **Swine** | **Porcine epidemic diarrhea virus** |
| **KJ645700.1** | **2014/1/22** | **Mexico** | **Swine** | **Porcine epidemic diarrhea virus** |
| **KJ645701.1** | **2014/1/27** | **USA** | **Swine** | **Porcine epidemic diarrhea virus** |
| **KJ645702.1** | **2014/1/29** | **USA** | **Swine** | **Porcine epidemic diarrhea virus** |
| **KJ645703.1** | **2014/2/2** | **USA** | **Swine** | **Porcine epidemic diarrhea virus** |
| **KJ645704.1** | **2013/6/26** | **USA** | **Swine** | **Porcine epidemic diarrhea virus** |
| **KJ645705.1** | **2013/11/18** | **USA** | **Swine** | **Porcine epidemic diarrhea virus** |
| **KJ645706.1** | **2013/11/25** | **USA** | **Swine** | **Porcine epidemic diarrhea virus** |
| **KJ645707.1** | **2013/12/3** | **USA** | **Swine** | **Porcine epidemic diarrhea virus** |
| **KJ645708.1** | **2013/11/3** | **Mexico** | **Swine** | **Porcine epidemic diarrhea virus** |
| **KJ662670.1** | **2013/12/1** | **South Korea** | **Swine** | **Porcine epidemic diarrhea virus** |
| **KJ769231.1** | **2014/3/1** | **USA** | **Swine** | **Swine deltacoronavirus OhioCVM1/2014** |
| **KJ777677.1** | **2012/2/1** | **China** | **Swine** | **Porcine epidemic diarrhea virus** |
| **KJ777678.1** | **2012/2/1** | **China** | **Swine** | **Porcine epidemic diarrhea virus** |
| **KJ778615.1** | **2013/5/20** | **USA** | **Swine** | **Porcine epidemic diarrhea virus** |
| **KJ778616.1** | **2013/5/20** | **USA** | **Swine** | **Porcine epidemic diarrhea virus** |
| **KJ960178.1** | **2013** | **Viet Nam** | **Swine** | **Porcine epidemic diarrhea virus** |
| **KJ960179.1** | **2013** | **Viet Nam** | **Swine** | **Porcine epidemic diarrhea virus** |
| **KJ960180.1** | **2013** | **Viet Nam** | **Swine** | **Porcine epidemic diarrhea virus** |
| **KM012168.1** | **2014/3/17** | **USA** | **Swine** | **Porcine coronavirus HKU15** |
| **KM052365.1** | **2013/5/15** | **USA** | **Swine** | **Porcine epidemic diarrhea virus** |
| **KM077139.1** | **2014/1/22** | **USA** | **Swine** | **Porcine epidemic diarrhea virus** |
| **KM089829.1** | **2012/11/10** | **China** | **Swine** | **Porcine epidemic diarrhea virus** |
| **KM242131.1** | **2014/3/1** | **China** | **Swine** | **Porcine epidemic diarrhea virus** |
| **KM392224.1** | **2013/6/1** | **USA** | **Swine** | **Porcine epidemic diarrhea virus** |
| **KM392225.1** | **2013/6/1** | **USA** | **Swine** | **Porcine epidemic diarrhea virus** |
| **KM392226.1** | **2013/11/1** | **USA** | **Swine** | **Porcine epidemic diarrhea virus** |
| **KM392227.1** | **2013/11/1** | **USA** | **Swine** | **Porcine epidemic diarrhea virus** |
| **KM392228.1** | **2013/12/1** | **USA** | **Swine** | **Porcine epidemic diarrhea virus** |
| **KM392229.1** | **2013/12/1** | **USA** | **Swine** | **Porcine epidemic diarrhea virus** |
| **KM392230.1** | **2013/12/1** | **USA** | **Swine** | **Porcine epidemic diarrhea virus** |
| **KM392231.1** | **2013/12/1** | **USA** | **Swine** | **Porcine epidemic diarrhea virus** |
| **KM392232.1** | **2013/12/1** | **USA** | **Swine** | **Porcine epidemic diarrhea virus** |
| **KM403155.1** | **2014/3/1** | **South Korea** | **Swine** | **Porcine epidemic diarrhea virus** |
| **KM609203.1** | **2012/3/1** | **China** | **Swine** | **Porcine epidemic diarrhea virus** |
| **KM609204.1** | **2011/6/1** | **China** | **Swine** | **Porcine epidemic diarrhea virus** |
| **KM609205.1** | **N/A** | **China** | **Swine** | **Porcine epidemic diarrhea virus** |
| **KM609206.1** | **2012/1/1** | **China** | **Swine** | **Porcine epidemic diarrhea virus** |
| **KM609207.1** | **2011/4/1** | **China** | **Swine** | **Porcine epidemic diarrhea virus** |
| **KM609208.1** | **2012/3/1** | **China** | **Swine** | **Porcine epidemic diarrhea virus** |
| **KM609209.1** | **2013/3/1** | **China** | **Swine** | **Porcine epidemic diarrhea virus** |
| **KM609210.1** | **2014/1/1** | **China** | **Swine** | **Porcine epidemic diarrhea virus** |
| **KM609211.1** | **2014/1/1** | **China** | **Swine** | **Porcine epidemic diarrhea virus** |
| **KM609212.1** | **2014/3/1** | **China** | **Swine** | **Porcine epidemic diarrhea virus** |
| **KM609213.1** | **2014/3/1** | **China** | **Swine** | **Porcine epidemic diarrhea virus** |
| **KM820765.1** | **2014/4/1** | **South Korea** | **Swine** | **Porcine deltacoronavirus KNU14-04** |
| **KM887144.1** | **2013** | **China** | **Swine** | **Porcine epidemic diarrhea virus** |
| **KM975735.1** | **2013/9/12** | **USA** | **Swine** | **Porcine epidemic diarrhea virus** |
| **KM975736.1** | **2013/12/9** | **USA** | **Swine** | **Porcine epidemic diarrhea virus** |
| **KM975737.1** | **2013/12/10** | **USA** | **Swine** | **Porcine epidemic diarrhea virus** |
| **KM975738.1** | **2013/5/16** | **USA** | **Swine** | **Porcine epidemic diarrhea virus** |
| **KM975739.1** | **2013/5/29** | **USA** | **Swine** | **Porcine epidemic diarrhea virus** |
| **KM975740.1** | **2013/12/31** | **USA** | **Swine** | **Porcine epidemic diarrhea virus** |
| **KM975741.1** | **2014/1/20** | **USA** | **Swine** | **Porcine epidemic diarrhea virus** |
| **KP162057.1** | **2014/3/15** | **China** | **Swine** | **Porcine epidemic diarrhea virus** |
| **KP202365.1** | **2014/8/29** | **USA** | **Swine** | **Porcine epidemic diarrhea virus** |
| **KP202848.1** | **2013/3/7** | **China** | **Swine** | **Transmissible gastroenteritis virus** |
| **KP403802.1** | **N/A** | **China** | **Swine** | **Porcine epidemic diarrhea virus** |
| **KP403954.1** | **2014/11/22** | **Ukraine** | **Swine** | **Porcine epidemic diarrhea virus** |
| **KP641661.1** | **2014/4/22** | **USA** | **Swine** | **Porcine epidemic diarrhea virus** |
| **KP641662.1** | **2014/10/14** | **USA** | **Swine** | **Porcine epidemic diarrhea virus** |
| **KP641663.1** | **2014/10/19** | **USA** | **Swine** | **Porcine epidemic diarrhea virus** |
| **KP688354.1** | **2014/11/13** | **USA** | **Swine** | **Porcine epidemic diarrhea virus** |
| **KP728470.1** | **2014/3/5** | **China** | **Swine** | **Porcine epidemic diarrhea virus** |
| **KP757890.1** | **2004/5/24** | **China** | **Swine** | **Porcine deltacoronavirus** |
| **KP757891.1** | **2014/12/26** | **China** | **Swine** | **Porcine deltacoronavirus** |
| **KP757892.1** | **2014/12/20** | **China** | **Swine** | **Porcine deltacoronavirus** |
| **KP765609.1** | **2013/7/1** | **China** | **Swine** | **Porcine epidemic diarrhea virus** |
| **KP890336.1** | **2014/12/1** | **China** | **Swine** | **Porcine epidemic diarrhea virus** |
| **KP981395.1** | **N/A** | **USA** | **Swine** | **Porcine deltacoronavirus** |
| **KR003452.1** | **2015/1/1** | **Belgium** | **Swine** | **Porcine epidemic diarrhea virus** |
| **KR011756.1** | **2014/12/1** | **France** | **Swine** | **Porcine epidemic diarrhea virus** |
| **KR061458.1** | **2009/1/14** | **Italy** | **Swine** | **Porcine epidemic diarrhea virus** |
| **KR061459.1** | **2009/9/4** | **Italy** | **Swine** | **Swine enteric coronavirus** |
| **KR078299.1** | **2013/6/1** | **USA** | **Swine** | **Porcine epidemic diarrhea virus** |
| **KR078300.1** | **2013/12/1** | **USA** | **Swine** | **Porcine epidemic diarrhea virus** |
| **KR095279.1** | **2015/1/27** | **China** | **Swine** | **Porcine epidemic diarrhea virus** |
| **KR131621.1** | **2015/3/1** | **China** | **Swine** | **Porcine deltacoronavirus** |
| **KR150443.1** | **2015/3/24** | **USA** | **Swine** | **Porcine deltacoronavirus** |
| **KR153325.1** | **2014/1/1** | **China** | **Swine** | **Porcine epidemic diarrhea virus** |
| **KR153326.1** | **2014/1/4** | **China** | **Swine** | **Porcine epidemic diarrhea virus** |
| **KR265759.1** | **2014/1/14** | **USA** | **Swine** | **Porcine epidemic diarrhea virus** |
| **KR265760.1** | **2014/2/12** | **USA** | **Swine** | **Porcine epidemic diarrhea virus** |
| **KR265761.1** | **2014/11/13** | **USA** | **Swine** | **Porcine epidemic diarrhea virus** |
| **KR265762.1** | **2014/12/12** | **USA** | **Swine** | **Porcine epidemic diarrhea virus** |
| **KR265763.1** | **2014/1/3** | **USA** | **Swine** | **Porcine epidemic diarrhea virus** |
| **KR265764.1** | **2014/2/18** | **USA** | **Swine** | **Porcine epidemic diarrhea virus** |
| **KR265765.1** | **2014/4/2** | **USA** | **Swine** | **Porcine epidemic diarrhea virus** |
| **KR265766.1** | **2014/4/24** | **Mexico** | **Swine** | **Porcine epidemic diarrhea virus** |
| **KR265767.1** | **2014/10/31** | **USA** | **Swine** | **Porcine epidemic diarrhea virus** |
| **KR265768.1** | **2014/2/11** | **USA** | **Swine** | **Porcine epidemic diarrhea virus** |
| **KR265769.1** | **2014/3/4** | **USA** | **Swine** | **Porcine epidemic diarrhea virus** |
| **KR265770.1** | **2014/3/11** | **USA** | **Swine** | **Porcine epidemic diarrhea virus** |
| **KR265771.1** | **2014/8/18** | **USA** | **Swine** | **Porcine epidemic diarrhea virus** |
| **KR265772.1** | **2014/1/7** | **USA** | **Swine** | **Porcine epidemic diarrhea virus** |
| **KR265773.1** | **2014/3/27** | **USA** | **Swine** | **Porcine epidemic diarrhea virus** |
| **KR265774.1** | **2014/5/16** | **USA** | **Swine** | **Porcine epidemic diarrhea virus** |
| **KR265775.1** | **2014/1/9** | **USA** | **Swine** | **Porcine epidemic diarrhea virus** |
| **KR265776.1** | **2014/2/3** | **USA** | **Swine** | **Porcine epidemic diarrhea virus** |
| **KR265777.1** | **2014/3/13** | **USA** | **Swine** | **Porcine epidemic diarrhea virus** |
| **KR265778.1** | **2014/3/14** | **USA** | **Swine** | **Porcine epidemic diarrhea virus** |
| **KR265779.1** | **2014/1/14** | **USA** | **Swine** | **Porcine epidemic diarrhea virus** |
| **KR265780.1** | **2014/1/17** | **USA** | **Swine** | **Porcine epidemic diarrhea virus** |
| **KR265781.1** | **2014/2/25** | **USA** | **Swine** | **Porcine epidemic diarrhea virus** |
| **KR265782.1** | **2014/4/16** | **USA** | **Swine** | **Porcine epidemic diarrhea virus** |
| **KR265783.1** | **2014/2/14** | **USA** | **Swine** | **Porcine epidemic diarrhea virus** |
| **KR265784.1** | **2014/2/25** | **USA** | **Swine** | **Porcine epidemic diarrhea virus** |
| **KR265785.1** | **2014/3/13** | **USA** | **Swine** | **Porcine epidemic diarrhea virus** |
| **KR265786.1** | **2014/3/14** | **USA** | **Swine** | **Porcine epidemic diarrhea virus** |
| **KR265787.1** | **2014/4/2** | **USA** | **Swine** | **Porcine epidemic diarrhea virus** |
| **KR265788.1** | **2014/4/8** | **USA** | **Swine** | **Porcine epidemic diarrhea virus** |
| **KR265789.1** | **2014/4/16** | **USA** | **Swine** | **Porcine epidemic diarrhea virus** |
| **KR265790.1** | **2014/10/14** | **USA** | **Swine** | **Porcine epidemic diarrhea virus** |
| **KR265791.1** | **2014/1/29** | **USA** | **Swine** | **Porcine epidemic diarrhea virus** |
| **KR265792.1** | **2014/9/26** | **USA** | **Swine** | **Porcine epidemic diarrhea virus** |
| **KR265793.1** | **2014/2/13** | **USA** | **Swine** | **Porcine epidemic diarrhea virus** |
| **KR265794.1** | **2014/3/4** | **USA** | **Swine** | **Porcine epidemic diarrhea virus** |
| **KR265795.1** | **2014/3/7** | **USA** | **Swine** | **Porcine epidemic diarrhea virus** |
| **KR265796.1** | **2014/3/19** | **USA** | **Swine** | **Porcine epidemic diarrhea virus** |
| **KR265797.1** | **2014/3/27** | **USA** | **Swine** | **Porcine epidemic diarrhea virus** |
| **KR265798.1** | **2014/4/2** | **USA** | **Swine** | **Porcine epidemic diarrhea virus** |
| **KR265799.1** | **2014/2/12** | **USA** | **Swine** | **Porcine epidemic diarrhea virus** |
| **KR265800.1** | **2014/3/4** | **USA** | **Swine** | **Porcine epidemic diarrhea virus** |
| **KR265801.1** | **2014/3/12** | **USA** | **Swine** | **Porcine epidemic diarrhea virus** |
| **KR265802.1** | **2014/3/14** | **USA** | **Swine** | **Porcine epidemic diarrhea virus** |
| **KR265803.1** | **2014/4/2** | **USA** | **Swine** | **Porcine epidemic diarrhea virus** |
| **KR265804.1** | **2014/5/14** | **USA** | **Swine** | **Porcine epidemic diarrhea virus** |
| **KR265805.1** | **2014/2/17** | **USA** | **Swine** | **Porcine epidemic diarrhea virus** |
| **KR265806.1** | **2014/4/7** | **USA** | **Swine** | **Porcine epidemic diarrhea virus** |
| **KR265807.1** | **2014/2/17** | **USA** | **Swine** | **Porcine epidemic diarrhea virus** |
| **KR265808.1** | **2014/2/17** | **USA** | **Swine** | **Porcine epidemic diarrhea virus** |
| **KR265809.1** | **2014/3/25** | **USA** | **Swine** | **Porcine epidemic diarrhea virus** |
| **KR265810.1** | **2014/4/24** | **USA** | **Swine** | **Porcine epidemic diarrhea virus** |
| **KR265811.1** | **2014/4/30** | **USA** | **Swine** | **Porcine epidemic diarrhea virus** |
| **KR265812.1** | **2014/9/30** | **USA** | **Swine** | **Porcine epidemic diarrhea virus** |
| **KR265813.1** | **2014/2/20** | **USA** | **Swine** | **Porcine epidemic diarrhea virus** |
| **KR265814.1** | **2014/2/28** | **USA** | **Swine** | **Porcine epidemic diarrhea virus** |
| **KR265815.1** | **2014/3/3** | **USA** | **Swine** | **Porcine epidemic diarrhea virus** |
| **KR265816.1** | **2014/3/10** | **USA** | **Swine** | **Porcine epidemic diarrhea virus** |
| **KR265817.1** | **2014/3/11** | **USA** | **Swine** | **Porcine epidemic diarrhea virus** |
| **KR265818.1** | **2014/4/3** | **USA** | **Swine** | **Porcine epidemic diarrhea virus** |
| **KR265819.1** | **2014/10/30** | **USA** | **Swine** | **Porcine epidemic diarrhea virus** |
| **KR265820.1** | **2014/5/7** | **USA** | **Swine** | **Porcine epidemic diarrhea virus** |
| **KR265821.1** | **2014/2/26** | **USA** | **Swine** | **Porcine epidemic diarrhea virus** |
| **KR265822.1** | **2014/3/4** | **USA** | **Swine** | **Porcine epidemic diarrhea virus** |
| **KR265823.1** | **2014/3/11** | **USA** | **Swine** | **Porcine epidemic diarrhea virus** |
| **KR265824.1** | **2014/12/4** | **USA** | **Swine** | **Porcine epidemic diarrhea virus** |
| **KR265825.1** | **2014/3/5** | **USA** | **Swine** | **Porcine epidemic diarrhea virus** |
| **KR265826.1** | **2014/3/13** | **USA** | **Swine** | **Porcine epidemic diarrhea virus** |
| **KR265827.1** | **2014/4/7** | **USA** | **Swine** | **Porcine epidemic diarrhea virus** |
| **KR265828.1** | **2014/3/24** | **USA** | **Swine** | **Porcine epidemic diarrhea virus** |
| **KR265829.1** | **2014/4/17** | **USA** | **Swine** | **Porcine epidemic diarrhea virus** |
| **KR265830.1** | **2014/4/3** | **USA** | **Swine** | **Porcine epidemic diarrhea virus** |
| **KR265831.1** | **2014/5/14** | **Canada** | **Swine** | **Porcine epidemic diarrhea virus** |
| **KR265832.1** | **2014/6/10** | **USA** | **Swine** | **Porcine epidemic diarrhea virus** |
| **KR265833.1** | **2014/12/12** | **USA** | **Swine** | **Porcine epidemic diarrhea virus** |
| **KR265834.1** | **2014/11/18** | **USA** | **Swine** | **Porcine epidemic diarrhea virus** |
| **KR265840.1** | **2014/1/22** | **USA** | **Swine** | **Porcine epidemic diarrhea virus** |
| **KR265841.1** | **2014/8/28** | **USA** | **Swine** | **Porcine epidemic diarrhea virus** |
| **KR265842.1** | **2014/8/28** | **USA** | **Swine** | **Porcine epidemic diarrhea virus** |
| **KR265843.1** | **2014/5/23** | **USA** | **Swine** | **Porcine epidemic diarrhea virus** |
| **KR265844.1** | **2014/5/30** | **USA** | **Swine** | **Porcine epidemic diarrhea virus** |
| **KR265845.1** | **2014/12/4** | **USA** | **Swine** | **Porcine epidemic diarrhea virus** |
| **KR265846.1** | **2014/2/18** | **USA** | **Swine** | **Porcine epidemic diarrhea virus** |
| **KR265847.1** | **2014/3/6** | **USA** | **Swine** | **Porcine deltacoronavirus** |
| **KR265848.1** | **2014/3/14** | **USA** | **Swine** | **Porcine deltacoronavirus** |
| **KR265849.1** | **2014/4/2** | **USA** | **Swine** | **Porcine deltacoronavirus** |
| **KR265850.1** | **2014/4/2** | **USA** | **Swine** | **Porcine deltacoronavirus** |
| **KR265851.1** | **2014/5/13** | **USA** | **Swine** | **Porcine deltacoronavirus** |
| **KR265852.1** | **2014/4/21** | **USA** | **Swine** | **Porcine deltacoronavirus** |
| **KR265853.1** | **2013/10/14** | **USA** | **Swine** | **Porcine deltacoronavirus** |
| **KR265854.1** | **2014/5/21** | **USA** | **Swine** | **Porcine deltacoronavirus** |
| **KR265855.1** | **2014/5/21** | **USA** | **Swine** | **Porcine deltacoronavirus** |
| **KR265856.1** | **2014/2/23** | **USA** | **Swine** | **Porcine deltacoronavirus** |
| **KR265857.1** | **2014/2/23** | **USA** | **Swine** | **Porcine deltacoronavirus** |
| **KR265858.1** | **2014/5/6** | **USA** | **Swine** | **Porcine deltacoronavirus** |
| **KR265859.1** | **2014/2/11** | **USA** | **Swine** | **Porcine deltacoronavirus** |
| **KR265860.1** | **2014/2/5** | **USA** | **Swine** | **Porcine deltacoronavirus** |
| **KR265861.1** | **2014/2/5** | **USA** | **Swine** | **Porcine deltacoronavirus** |
| **KR265862.1** | **2014/3/26** | **USA** | **Swine** | **Porcine deltacoronavirus** |
| **KR265863.1** | **2014/3/27** | **USA** | **Swine** | **Porcine deltacoronavirus** |
| **KR265864.1** | **2014/3/14** | **USA** | **Swine** | **Porcine deltacoronavirus** |
| **KR265865.1** | **2014/6/5** | **USA** | **Swine** | **Porcine deltacoronavirus** |
| **KR610991.1** | **2014/10/10** | **Thailand** | **Swine** | **Porcine epidemic diarrhea virus** |
| **KR610992.1** | **2014/10/10** | **Thailand** | **Swine** | **Porcine epidemic diarrhea virus** |
| **KR610993.1** | **2014/7/15** | **Thailand** | **Swine** | **Porcine epidemic diarrhea virus** |
| **KR610994.1** | **2014/7/15** | **Thailand** | **Swine** | **Porcine epidemic diarrhea virus** |
| **KR809885.1** | **2015/3/18** | **China** | **Swine** | **Porcine epidemic diarrhea virus** |
| **KR818832.1** | **2013/2/1** | **China** | **Swine** | **Porcine epidemic diarrhea virus** |
| **KR818833.1** | **N/A** | **China** | **Swine** | **Porcine epidemic diarrhea virus** |
| **KR873431.1** | **2014/11/1** | **South Korea** | **Swine** | **Porcine epidemic diarrhea virus** |
| **KR873434.1** | **2014/11/1** | **South Korea** | **Swine** | **Porcine epidemic diarrhea virus** |
| **KR873435.1** | **2014/11/1** | **South Korea** | **Swine** | **Porcine epidemic diarrhea virus** |
| **KT021227.1** | **2013/8/2** | **China** | **Swine** | **Porcine epidemic diarrhea virus** |
| **KT021228.1** | **2013/10/5** | **China** | **Swine** | **Porcine epidemic diarrhea virus** |
| **KT021229.1** | **2013/11/8** | **China** | **Swine** | **Porcine epidemic diarrhea virus** |
| **KT021230.1** | **2014/2/5** | **China** | **Swine** | **Porcine epidemic diarrhea virus** |
| **KT021231.1** | **2014/5/10** | **China** | **Swine** | **Porcine epidemic diarrhea virus** |
| **KT021232.1** | **2014/8/8** | **China** | **Swine** | **Porcine epidemic diarrhea virus** |
| **KT021233.1** | **2014/11/11** | **China** | **Swine** | **Porcine epidemic diarrhea virus** |
| **KT021234.1** | **2015/3/20** | **China** | **Swine** | **Porcine deltacoronavirus** |
| **KT199103.1** | **2015/6/1** | **China** | **Swine** | **Porcine epidemic diarrhea virus** |
| **KT266822.1** | **2012** | **China** | **Swine** | **Porcine deltacoronavirus** |
| **KT323979.1** | **1998** | **China** | **Swine** | **Porcine epidemic diarrhea virus** |
| **KT323980.1** | **2014/9/11** | **China** | **Swine** | **Porcine epidemic diarrhea virus** |
| **KT381613.1** | **2014/5/7** | **USA** | **Swine** | **Porcine coronavirus HKU15** |
| **KT591944.1** | **2013/12/31** | **USA** | **Swine** | **Porcine epidemic diarrhea virus** |
| **KT696544.1** | **2012** | **China** | **Swine** | **Transmissible gastroenteritis virus** |
| **KT860508.1** | **2013/12/31** | **USA** | **Swine** | **Porcine epidemic diarrhea virus** |
| **KT941120.1** | **2014/10/15** | **Viet Nam** | **Swine** | **Porcine epidemic diarrhea virus** |
| **KU051641.1** | **2015/6/10** | **Thailand** | **Swine** | **Porcine deltacoronavirus** |
| **KU051649.1** | **2015/6/30** | **Thailand** | **Swine** | **Porcine deltacoronavirus** |
| **KU252649.1** | **2014/5/8** | **China** | **Swine** | **Porcine epidemic diarrhea virus** |
| **KU297956.1** | **2015/9/3** | **Slovenia** | **Swine** | **Porcine epidemic diarrhea virus** |
| **KU380331.1** | **2015/5/1** | **China** | **Swine** | **Porcine epidemic diarrhea virus** |
| **KU558701.1** | **2013/11/14** | **China** | **Swine** | **Porcine epidemic diarrhea virus** |
| **KU558702.1** | **2013/5/29** | **USA** | **Swine** | **Porcine epidemic diarrhea virus** |
| **KU569509.1** | **2014/5/2** | **Colombia** | **Swine** | **Porcine epidemic diarrhea virus** |
| **KU646831.1** | **2012** | **China** | **Swine** | **Porcine epidemic diarrhea virus** |
| **KU664503.1** | **2013/11/14** | **China** | **Swine** | **Porcine epidemic diarrhea virus** |
| **KU665558.1** | **2014/6/26** | **China** | **Swine** | **Porcine deltacoronavirus** |
| **KU729220.1** | **1998/2/10** | **China** | **Swine** | **Transmissible gastroenteritis virus** |
| **KU847996.1** | **2015/7/26** | **China** | **Swine** | **Porcine epidemic diarrhea virus** |
| **KU893861.1** | **2013/6/1** | **USA** | **Swine** | **Porcine epidemic diarrhea virus** |
| **KU893862.1** | **2013/10/1** | **USA** | **Swine** | **Porcine epidemic diarrhea virus** |
| **KU893863.1** | **2014/1/1** | **USA** | **Swine** | **Porcine epidemic diarrhea virus** |
| **KU893864.1** | **2014/3/1** | **USA** | **Swine** | **Porcine epidemic diarrhea virus** |
| **KU893865.1** | **2014/3/1** | **USA** | **Swine** | **Porcine epidemic diarrhea virus** |
| **KU893866.1** | **2014/6/1** | **USA** | **Swine** | **Porcine epidemic diarrhea virus** |
| **KU893867.1** | **2014/8/1** | **USA** | **Swine** | **Porcine epidemic diarrhea virus** |
| **KU893868.1** | **2014/8/1** | **USA** | **Swine** | **Porcine epidemic diarrhea virus** |
| **KU893869.1** | **2015/6/1** | **USA** | **Swine** | **Porcine epidemic diarrhea virus** |
| **KU893870.1** | **2015/6/1** | **USA** | **Swine** | **Porcine epidemic diarrhea virus** |
| **KU893871.1** | **2015/6/1** | **USA** | **Swine** | **Porcine epidemic diarrhea virus** |
| **KU893872.1** | **2015/10/1** | **USA** | **Swine** | **Porcine epidemic diarrhea virus** |
| **KU893873.1** | **2015/10/1** | **USA** | **Swine** | **Porcine epidemic diarrhea virus** |
| **KU975389.1** | **2014/11/1** | **China** | **Swine** | **Porcine epidemic diarrhea virus** |
| **KU981059.1** | **2015/2/16** | **China** | **Swine** | **Porcine deltacoronavirus** |
| **KU981060.1** | **2015/4/15** | **China** | **Swine** | **Porcine deltacoronavirus** |
| **KU981061.1** | **2015/7/18** | **China** | **Swine** | **Porcine deltacoronavirus** |
| **KU981062.1** | **2015/12/30** | **China** | **Swine** | **Porcine deltacoronavirus** |
| **KU982966.1** | **2015/10/22** | **USA** | **Swine** | **Porcine epidemic diarrhea virus** |
| **KU982967.1** | **2015/10/21** | **USA** | **Swine** | **Porcine epidemic diarrhea virus** |
| **KU982968.1** | **2015/11/6** | **USA** | **Swine** | **Porcine epidemic diarrhea virus** |
| **KU982969.1** | **2015/10/21** | **USA** | **Swine** | **Porcine epidemic diarrhea virus** |
| **KU982970.1** | **2015/11/6** | **USA** | **Swine** | **Porcine epidemic diarrhea virus** |
| **KU982971.1** | **2015/1/9** | **USA** | **Swine** | **Porcine epidemic diarrhea virus** |
| **KU982972.1** | **2015/10/22** | **USA** | **Swine** | **Porcine epidemic diarrhea virus** |
| **KU982973.1** | **2015/11/12** | **USA** | **Swine** | **Porcine epidemic diarrhea virus** |
| **KU982974.1** | **2015/10/6** | **USA** | **Swine** | **Porcine epidemic diarrhea virus** |
| **KU982975.1** | **2015/10/26** | **USA** | **Swine** | **Porcine epidemic diarrhea virus** |
| **KU982976.1** | **2015/10/22** | **USA** | **Swine** | **Porcine epidemic diarrhea virus** |
| **KU982977.1** | **2015/11/5** | **USA** | **Swine** | **Porcine epidemic diarrhea virus** |
| **KU982978.1** | **2015/12/4** | **USA** | **Swine** | **Porcine epidemic diarrhea virus** |
| **KU982979.1** | **2015/11/4** | **USA** | **Swine** | **Porcine epidemic diarrhea virus** |
| **KU982980.1** | **2015/10/20** | **USA** | **Swine** | **Porcine epidemic diarrhea virus** |
| **KU982981.1** | **2015/10/19** | **USA** | **Swine** | **Porcine epidemic diarrhea virus** |
| **KU984334.1** | **2015/11/1** | **Thailand** | **Swine** | **Porcine deltacoronavirus** |
| **KX016034.1** | **2014/1/4** | **China** | **Swine** | **Porcine epidemic diarrhea virus** |
| **KX022602.1** | **2015/10/15** | **USA** | **Swine** | **Porcine deltacoronavirus** |
| **KX022603.1** | **2015/12/18** | **USA** | **Swine** | **Porcine deltacoronavirus** |
| **KX022604.1** | **2015/11/27** | **USA** | **Swine** | **Porcine deltacoronavirus** |
| **KX022605.1** | **2015/12/21** | **USA** | **Swine** | **Porcine deltacoronavirus** |
| **KX058031.1** | **2014/12/5** | **China** | **Swine** | **Porcine epidemic diarrhea virus** |
| **KX058032.1** | **2014/12/5** | **China** | **Swine** | **Porcine epidemic diarrhea virus** |
| **KX058033.1** | **2014/12/5** | **China** | **Swine** | **Porcine epidemic diarrhea virus** |
| **KX058075.1** | **2012** | **China** | **Swine** | **Transmissible gastroenteritis virus** |
| **KX064280.1** | **2014/10/20** | **China** | **Swine** | **Porcine epidemic diarrhea virus** |
| **KX066126.1** | **2011/11/1** | **China** | **Swine** | **Porcine epidemic diarrhea virus** |
| **KX083668.1** | **2015** | **China** | **Swine** | **Transmissible gastroenteritis virus** |
| **KX118627.1** | **2016/1/20** | **Laos** | **Swine** | **Porcine deltacoronavirus** |
| **KX289955.1** | **2016/3/1** | **Hungary** | **Swine** | **Porcine epidemic diarrhea virus** |
| **KX361343.1** | **2013/2/1** | **Thailand** | **Swine** | **Porcine deltacoronavirus** |
| **KX361344.1** | **2013/3/1** | **Thailand** | **Swine** | **Porcine deltacoronavirus** |
| **KX361345.1** | **2015/12/1** | **Thailand** | **Swine** | **Porcine deltacoronavirus** |
| **KX443143.2** | **2016** | **China** | **Swine** | **Porcine deltacoronavirus** |
| **KX499468.1** | **2015/12/22** | **China** | **Swine** | **Transmissible gastroenteritis virus** |
| **KX534205.1** | **2015/1/25** | **China** | **Swine** | **Porcine epidemic diarrhea virus** |
| **KX534206.1** | **2015/1/5** | **China** | **Swine** | **Porcine epidemic diarrhea virus** |
| **KX550281.1** | **2015/1/16** | **China** | **Swine** | **Porcine epidemic diarrhea virus** |
| **KX580953.1** | **2013** | **China** | **Swine** | **Porcine epidemic diarrhea virus** |
| **KX580958.1** | **2016/7/7** | **USA** | **Swine** | **Porcine epidemic diarrhea virus** |
| **KX683006.1** | **2013/6/1** | **N/A** | **Swine** | **Porcine epidemic diarrhea virus** |
| **KX791060.1** | **2014/5/12** | **China** | **Swine** | **Porcine epidemic diarrhea virus** |
| **KX812523.1** | **2016/3/30** | **China** | **Swine** | **Porcine epidemic diarrhea virus** |
| **KX812524.1** | **2016/8/1** | **China** | **Swine** | **Porcine epidemic diarrhea virus** |
| **KX834351.1** | **2015/10/10** | **Viet Nam** | **Swine** | **Porcine deltacoronavirus** |
| **KX834352.1** | **2015/12/8** | **Viet Nam** | **Swine** | **Porcine deltacoronavirus** |
| **KX839246.1** | **2013/5/1** | **China** | **Swine** | **Porcine epidemic diarrhea virus** |
| **KX839247.1** | **2015/9/1** | **China** | **Swine** | **Porcine epidemic diarrhea virus** |
| **KX839248.1** | **2015/9/1** | **China** | **Swine** | **Porcine epidemic diarrhea virus** |
| **KX839249.1** | **2015/9/1** | **China** | **Swine** | **Porcine epidemic diarrhea virus** |
| **KX839250.1** | **2015/9/1** | **China** | **Swine** | **Porcine epidemic diarrhea virus** |
| **KX839251.1** | **2015/9/1** | **China** | **Swine** | **Porcine epidemic diarrhea virus** |
| **KX883635.1** | **2014** | **China** | **Swine** | **Porcine epidemic diarrhea virus** |
| **KX900393.1** | **2006/12/8** | **USA** | **Swine** | **Transmissible gastroenteritis virus** |
| **KX900394.1** | **1988** | **USA** | **Swine** | **Transmissible gastroenteritis virus** |
| **KX900395.1** | **2006/11/8** | **USA** | **Swine** | **Transmissible gastroenteritis virus** |
| **KX900396.1** | **2006/11/17** | **USA** | **Swine** | **Transmissible gastroenteritis virus** |
| **KX900397.1** | **2007/2/8** | **USA** | **Swine** | **Transmissible gastroenteritis virus** |
| **KX900398.1** | **2007/2/8** | **USA** | **Swine** | **Transmissible gastroenteritis virus** |
| **KX900399.1** | **2007/2/16** | **USA** | **Swine** | **Transmissible gastroenteritis virus** |
| **KX900400.1** | **2008/3/6** | **USA** | **Swine** | **Transmissible gastroenteritis virus** |
| **KX900401.1** | **2008/4/15** | **USAe** | **Swine** | **Transmissible gastroenteritis virus** |
| **KX900402.1** | **2008/4/17** | **Mexico** | **Swine** | **Transmissible gastroenteritis virus** |
| **KX900403.1** | **2008/4/9** | **USA** | **Swine** | **Transmissible gastroenteritis virus** |
| **KX900404.1** | **2012/11/29** | **USA** | **Swine** | **Transmissible gastroenteritis virus** |
| **KX900405.1** | **2013/1/4** | **USA** | **Swine** | **Transmissible gastroenteritis virus** |
| **KX900406.1** | **2013/2/28** | **USA** | **Swine** | **Transmissible gastroenteritis virus** |
| **KX900407.1** | **2013/3/1** | **USA** | **Swine** | **Transmissible gastroenteritis virus** |
| **KX900408.1** | **2014/1/17** | **USA** | **Swine** | **Transmissible gastroenteritis virus** |
| **KX900409.1** | **2014/1/31** | **USA** | **Swine** | **Transmissible gastroenteritis virus** |
| **KX900410.1** | **2014/2/4** | **USA** | **Swine** | **Transmissible gastroenteritis virus** |
| **KX900411.1** | **2014/2/5** | **USA** | **Swine** | **Transmissible gastroenteritis virus** |
| **KX981440.1** | **2016/5/1** | **China** | **Swine** | **Porcine epidemic diarrhea virus** |
| **KX998969.1** | **2015/12/1** | **Viet Nam** | **Swine** | **Porcine deltacoronavirus** |
| **KY007139.1** | **2015/5/11** | **China** | **Swine** | **Porcine epidemic diarrhea virus** |
| **KY007140.1** | **2015/3/2** | **China** | **Swine** | **Porcine epidemic diarrhea virus** |
| **KY019623.1** | **2015/11/3** | **Slovenia** | **Swine** | **Porcine epidemic diarrhea virus** |
| **KY019624.1** | **2015/11/3** | **Slovenia** | **Swine** | **Porcine epidemic diarrhea virus** |
| **KY065120.1** | **2016** | **China** | **Swine** | **Porcine deltacoronavirus** |
| **KY070587.1** | **2016** | **China** | **Swine** | **Porcine epidemic diarrhea virus** |
| **KY111278.1** | **2016/1/28** | **Italy** | **Swine** | **Porcine epidemic diarrhea virus** |
| **KY293677.1** | **2016/5/23** | **China** | **Swine** | **Porcine deltacoronavirus** |
| **KY293678.1** | **2016/5/23** | **China** | **Swine** | **Porcine deltacoronavirus** |
| **KY354363.1** | **2016/4/1** | **South Korea** | **Swine** | **Porcine deltacoronavirus** |
| **KY354364.1** | **2016/4/1** | **South Korea** | **Swine** | **Porcine deltacoronavirus** |
| **KY363867.1** | **2016/3/18** | **China** | **Swine** | **Porcine deltacoronavirus** |
| **KY363868.1** | **2016/1/5** | **China** | **Swine** | **Porcine deltacoronavirus** |
| **KY364365.1** | **2014/7/1** | **South Korea** | **Swine** | **Porcine deltacoronavirus** |
| **KY420075.1** | **2015/3/14** | **China** | **Swine** | **Porcine epidemic diarrhea virus** |
| **KY486713.1** | **2015/9/1** | **China** | **Swine** | **Porcine epidemic diarrhea virus** |
| **KY486714.1** | **2015/9/1** | **China** | **Swine** | **Porcine epidemic diarrhea virus** |
| **KY499261.1** | **N/A** | **USA** | **Swine** | **Porcine epidemic diarrhea virus** |
| **KY499262.1** | **N/A** | **USA** | **Swine** | **Porcine epidemic diarrhea virus** |
| **KY513724.1** | **2014** | **China** | **Swine** | **Porcine deltacoronavirus** |
| **KY513725.1** | **2014** | **China** | **Swine** | **Porcine deltacoronavirus** |
| **KY649107.1** | **2016/5/1** | **China** | **Swine** | **Porcine epidemic diarrhea virus** |
| **KY793536.1** | **2015/2/14** | **China** | **Swine** | **Porcine epidemic diarrhea virus** |
| **KY825240.1** | **2016/1/1** | **South Korea** | **Swine** | **Porcine epidemic diarrhea virus** |
| **KY825241.1** | **2016/1/1** | **South Korea** | **Swine** | **Porcine epidemic diarrhea virus** |
| **KY825242.1** | **2016/1/1** | **South Korea** | **Swine** | **Porcine epidemic diarrhea virus** |
| **KY825243.1** | **2016/1/1** | **South Korea** | **Swine** | **Porcine epidemic diarrhea virus** |
| **KY926512.1** | **2016/11/1** | **South Korea** | **Swine** | **Porcine deltacoronavirus** |
| **KY928065.1** | **2016/3/1** | **China** | **Swine** | **Porcine epidemic diarrhea virus** |
| **KY929405.1** | **2015/5/4** | **Taiwan** | **Swine** | **Porcine epidemic diarrhea virus** |
| **KY929406.1** | **2016/1/2** | **Taiwan** | **Swine** | **Porcine epidemic diarrhea virus** |
| **KY963963.1** | **2016/3/1** | **South Korea** | **Swine** | **Porcine epidemic diarrhea virus** |
| **LC022792.1** | **2014/10/1** | **Japan** | **Swine** | **Porcine epidemic diarrhea virus** |
| **LC053455.1** | **2010/1/1** | **Thailand** | **Swine** | **Porcine epidemic diarrhea virus** |
| **LC063810.1** | **2014/2/1** | **Japan** | **Swine** | **Porcine epidemic diarrhea virus** |
| **LC063811.1** | **2014/3/1** | **Japan** | **Swine** | **Porcine epidemic diarrhea virus** |
| **LC063812.1** | **2014/4/1** | **Japan** | **Swine** | **Porcine epidemic diarrhea virus** |
| **LC063813.1** | **2014/4/1** | **Japan** | **Swine** | **Porcine epidemic diarrhea virus** |
| **LC063814.1** | **2013/12/1** | **Japan** | **Swine** | **Porcine epidemic diarrhea virus** |
| **LC063815.1** | **2013/12/1** | **Japan** | **Swine** | **Porcine epidemic diarrhea virus** |
| **LC063816.1** | **2014/2/1** | **Japan** | **Swine** | **Porcine epidemic diarrhea virus** |
| **LC063817.1** | **2014/1/1** | **Japan** | **Swine** | **Porcine epidemic diarrhea virus** |
| **LC063818.1** | **2014/1/1** | **Japan** | **Swine** | **Porcine epidemic diarrhea virus** |
| **LC063819.1** | **2014/4/1** | **Japan** | **Swine** | **Porcine epidemic diarrhea virus** |
| **LC063820.1** | **2013/11/1** | **Japan** | **Swine** | **Porcine epidemic diarrhea virus** |
| **LC063821.1** | **2013/11/1** | **Japan** | **Swine** | **Porcine epidemic diarrhea virus** |
| **LC063822.1** | **2014/3/1** | **Japan** | **Swine** | **Porcine epidemic diarrhea virus** |
| **LC063823.1** | **2014/4/1** | **Japan** | **Swine** | **Porcine epidemic diarrhea virus** |
| **LC063824.1** | **2014/4/1** | **Japan** | **Swine** | **Porcine epidemic diarrhea virus** |
| **LC063825.1** | **2014/5/1** | **Japan** | **Swine** | **Porcine epidemic diarrhea virus** |
| **LC063826.1** | **2014/4/1** | **Japan** | **Swine** | **Porcine epidemic diarrhea virus** |
| **LC063827.1** | **2014/3/1** | **Japan** | **Swine** | **Porcine epidemic diarrhea virus** |
| **LC063828.1** | **2014/10/1** | **Japan** | **Swine** | **Porcine epidemic diarrhea virus** |
| **LC063829.1** | **2014/4/1** | **Japan** | **Swine** | **Porcine epidemic diarrhea virus** |
| **LC063830.1** | **2014/4/1** | **Japan** | **Swine** | **Porcine epidemic diarrhea virus** |
| **LC063831.1** | **2014/5/1** | **Japan** | **Swine** | **Porcine epidemic diarrhea virus** |
| **LC063832.1** | **2014/5/1** | **Japan** | **Swine** | **Porcine epidemic diarrhea virus** |
| **LC063833.1** | **2014/4/1** | **Japan** | **Swine** | **Porcine epidemic diarrhea virus** |
| **LC063834.1** | **2014/4/1** | **Japan** | **Swine** | **Porcine epidemic diarrhea virus** |
| **LC063835.1** | **2014/4/1** | **Japan** | **Swine** | **Porcine epidemic diarrhea virus** |
| **LC063836.1** | **2013/10/1** | **Japan** | **Swine** | **Porcine epidemic diarrhea virus** |
| **LC063837.1** | **2014/4/1** | **Japan** | **Swine** | **Porcine epidemic diarrhea virus** |
| **LC063838.1** | **2014/7/1** | **Japan** | **Swine** | **Porcine epidemic diarrhea virus** |
| **LC063839.1** | **2014/4/1** | **Japan** | **Swine** | **Porcine epidemic diarrhea virus** |
| **LC063840.1** | **2014/4/1** | **Japan** | **Swine** | **Porcine epidemic diarrhea virus** |
| **LC063841.1** | **2014/4/1** | **Japan** | **Swine** | **Porcine epidemic diarrhea virus** |
| **LC063842.1** | **2014/4/1** | **Japan** | **Swine** | **Porcine epidemic diarrhea virus** |
| **LC063843.1** | **2014/5/1** | **Japan** | **Swine** | **Porcine epidemic diarrhea virus** |
| **LC063844.1** | **2014/3/1** | **Japan** | **Swine** | **Porcine epidemic diarrhea virus** |
| **LC063845.1** | **2014/3/1** | **Japan** | **Swine** | **Porcine epidemic diarrhea virus** |
| **LC063846.1** | **2013/12/1** | **Japan** | **Swine** | **Porcine epidemic diarrhea virus** |
| **LC063847.1** | **2014/3/1** | **Japan** | **Swine** | **Porcine epidemic diarrhea virus** |
| **LC216914.1** | **2014/2/13** | **Hong Kong** | **Swine** | **Coronavirus HKU15** |
| **LC216915.1** | **2014/2/13** | **Hong Kong** | **Swine** | **Coronavirus HKU15** |
| **LC260038.1** | **2014/5/1** | **Japan** | **Swine** | **Porcine deltacoronavirus** |
| **LC260039.1** | **2014/5/1** | **Japan** | **Swine** | **Porcine deltacoronavirus** |
| **LC260040.1** | **2014/5/1** | **Japan** | **Swine** | **Porcine deltacoronavirus** |
| **LC260041.1** | **2014/5/1** | **Japan** | **Swine** | **Porcine deltacoronavirus** |
| **LC260042.1** | **2014/3/1** | **Japan** | **Swine** | **Porcine deltacoronavirus** |
| **LC260043.1** | **2014/8/1** | **Japan** | **Swine** | **Porcine deltacoronavirus** |
| **LC260044.1** | **2014/12/1** | **Japan** | **Swine** | **Porcine deltacoronavirus** |
| **LC260045.1** | **2016/9/1** | **Japan** | **Swine** | **Porcine deltacoronavirus** |
| **LM645057.1** | **N/A** | **Germany** | **Swine** | **Porcine epidemic diarrhea virus** |
| **LM645058.1** | **N/A** | **Germany** | **Swine** | **Porcine epidemic diarrhea virus** |
| **LP731468.1** | **N/A** | **N/A** | **Swine** | **Porcine epidemic diarrhea virus** |
| **LR812926.1** | **N/A** | **N/A** | **Swine** | **Porcine epidemic diarrhea virus** |
| **LR812927.1** | **N/A** | **N/A** | **Swine** | **Porcine epidemic diarrhea virus** |
| **LR812928.1** | **N/A** | **N/A** | **Swine** | **Porcine epidemic diarrhea virus** |
| **LR812929.1** | **N/A** | **N/A** | **Swine** | **Porcine epidemic diarrhea virus** |
| **LR812930.1** | **N/A** | **N/A** | **Swine** | **Porcine epidemic diarrhea virus** |
| **LR812931.1** | **N/A** | **N/A** | **Swine** | **Porcine epidemic diarrhea virus** |
| **LR812932.1** | **N/A** | **N/A** | **Swine** | **Porcine epidemic diarrhea virus** |
| **LT545990.1** | **N/A** | **N/A** | **Swine** | **Swine enteric coronavirus** |
| **LT897799.1** | **N/A** | **N/A** | **Swine** | **Porcine epidemic diarrhea virus** |
| **LT898408.1** | **N/A** | **N/A** | **Swine** | **Porcine epidemic diarrhea virus** |
| **LT898409.1** | **N/A** | **N/A** | **Swine** | **Porcine epidemic diarrhea virus** |
| **LT898410.1** | **N/A** | **N/A** | **Swine** | **Porcine epidemic diarrhea virus** |
| **LT898411.1** | **N/A** | **N/A** | **Swine** | **Porcine epidemic diarrhea virus** |
| **LT898412.1** | **N/A** | **N/A** | **Swine** | **Porcine epidemic diarrhea virus** |
| **LT898413.1** | **N/A** | **N/A** | **Swine** | **Porcine epidemic diarrhea virus** |
| **LT898414.1** | **N/A** | **N/A** | **Swine** | **Porcine epidemic diarrhea virus** |
| **LT898415.1** | **N/A** | **N/A** | **Swine** | **Porcine epidemic diarrhea virus** |
| **LT898416.1** | **N/A** | **N/A** | **Swine** | **Porcine epidemic diarrhea virus** |
| **LT898417.1** | **N/A** | **N/A** | **Swine** | **Porcine epidemic diarrhea virus** |
| **LT898418.1** | **N/A** | **N/A** | **Swine** | **Porcine epidemic diarrhea virus** |
| **LT898420.1** | **N/A** | **N/A** | **Swine** | **Porcine epidemic diarrhea virus** |
| **LT898421.1** | **N/A** | **N/A** | **Swine** | **Porcine epidemic diarrhea virus** |
| **LT898423.1** | **N/A** | **N/A** | **Swine** | **Porcine epidemic diarrhea virus** |
| **LT898425.1** | **N/A** | **N/A** | **Swine** | **Porcine epidemic diarrhea virus** |
| **LT898426.1** | **N/A** | **N/A** | **Swine** | **Porcine epidemic diarrhea virus** |
| **LT898427.1** | **N/A** | **N/A** | **Swine** | **Porcine epidemic diarrhea virus** |
| **LT898430.1** | **N/A** | **N/A** | **Swine** | **Porcine epidemic diarrhea virus** |
| **LT898431.1** | **N/A** | **N/A** | **Swine** | **Porcine epidemic diarrhea virus** |
| **LT898432.1** | **N/A** | **N/A** | **Swine** | **Porcine epidemic diarrhea virus** |
| **LT898433.1** | **N/A** | **N/A** | **Swine** | **Porcine epidemic diarrhea virus** |
| **LT898435.1** | **N/A** | **N/A** | **Swine** | **Porcine epidemic diarrhea virus** |
| **LT898436.1** | **N/A** | **N/A** | **Swine** | **Porcine epidemic diarrhea virus** |
| **LT898438.1** | **N/A** | **N/A** | **Swine** | **Porcine epidemic diarrhea virus** |
| **LT898439.1** | **N/A** | **N/A** | **Swine** | **Porcine epidemic diarrhea virus** |
| **LT898440.1** | **N/A** | **N/A** | **Swine** | **Porcine epidemic diarrhea virus** |
| **LT898441.1** | **N/A** | **N/A** | **Swine** | **Porcine epidemic diarrhea virus** |
| **LT898443.1** | **N/A** | **N/A** | **Swine** | **Porcine epidemic diarrhea virus** |
| **LT898444.1** | **N/A** | **N/A** | **Swine** | **Porcine epidemic diarrhea virus** |
| **LT898445.1** | **N/A** | **N/A** | **Swine** | **Porcine epidemic diarrhea virus** |
| **LT898446.1** | **N/A** | **N/A** | **Swine** | **Porcine epidemic diarrhea virus** |
| **LT898447.1** | **N/A** | **N/A** | **Swine** | **Porcine epidemic diarrhea virus** |
| **LT900498.1** | **N/A** | **N/A** | **Swine** | **Porcine epidemic diarrhea virus** |
| **LT900499.1** | **N/A** | **N/A** | **Swine** | **Porcine epidemic diarrhea virus** |
| **LT900500.1** | **N/A** | **N/A** | **Swine** | **Porcine epidemic diarrhea virus** |
| **LT900501.1** | **N/A** | **N/A** | **Swine** | **Porcine epidemic diarrhea virus** |
| **LT900502.1** | **N/A** | **N/A** | **Swine** | **Porcine epidemic diarrhea virus** |
| **LT905450.1** | **N/A** | **N/A** | **Swine** | **Porcine epidemic diarrhea virus** |
| **LT905451.1** | **N/A** | **N/A** | **Swine** | **Porcine epidemic diarrhea virus** |
| **LT906581.1** | **N/A** | **N/A** | **Swine** | **Porcine epidemic diarrhea virus** |
| **LT906582.1** | **N/A** | **N/A** | **Swine** | **Porcine epidemic diarrhea virus** |
| **LT906620.1** | **N/A** | **N/A** | **Swine** | **Porcine epidemic diarrhea virus** |
| **MA347662.1** | **N/A** | **N/A** | **Swine** | **Porcine epidemic diarrhea virus** |
| **MF041982.1** | **2016/12/23** | **China** | **Swine** | **Porcine deltacoronavirus** |
| **MF094681.1** | **N/A** | **China** | **Swine** | **Swine acute diarrhea syndrome coronavirus** |
| **MF094682.1** | **N/A** | **China** | **Swine** | **Swine acute diarrhea syndrome coronavirus** |
| **MF094683.1** | **N/A** | **China** | **Swine** | **Swine acute diarrhea syndrome coronavirus** |
| **MF094684.1** | **N/A** | **China** | **Swine** | **Swine acute diarrhea syndrome coronavirus** |
| **MF094685.1** | **N/A** | **China** | **Swine** | **Swine acute diarrhea syndrome related coronavirus** |
| **MF094686.1** | **N/A** | **China** | **Swine** | **Swine acute diarrhea syndrome related coronavirus** |
| **MF094687.1** | **N/A** | **China** | **Swine** | **Swine acute diarrhea syndrome related coronavirus** |
| **MF094688.1** | **N/A** | **China** | **Swine** | **Swine acute diarrhea syndrome related coronavirus** |
| **MF095123.1** | **2017/2/15** | **China** | **Swine** | **Porcine deltacoronavirus** |
| **MF167434.1** | **2017/3/6** | **China** | **Swine** | **Porcine enteric alphacoronavirus GDS04** |
| **MF280390.1** | **2016** | **China** | **Swine** | **Porcine deltacoronavirus** |
| **MF281416.1** | **2017/2/21** | **South Korea** | **Swine** | **Porcine epidemic diarrhea virus** |
| **MF346935.1** | **2016/7/1** | **China** | **Swine** | **Porcine epidemic diarrhea virus** |
| **MF373643.1** | **2014** | **USA** | **Swine** | **Porcine epidemic diarrhea virus** |
| **MF375374.1** | **2017/5/21** | **China** | **Swine** | **Porcine epidemic diarrhea virus** |
| **MF431742.1** | **2015** | **China** | **Swine** | **Porcine deltacoronavirus** |
| **MF431743.1** | **2014** | **China** | **Swine** | **Porcine deltacoronavirus** |
| **MF462814.1** | **2016/9/10** | **China** | **Swine** | **Porcine epidemic diarrhea virus** |
| **MF577027.1** | **N/A** | **Russia** | **Swine** | **Porcine epidemic diarrhea virus** |
| **MF642322.1** | **2016/8/1** | **China** | **Swine** | **Porcine deltacoronavirus** |
| **MF642323.1** | **2016/8/1** | **China** | **Swine** | **Porcine deltacoronavirus** |
| **MF642324.1** | **2017/4/1** | **China** | **Swine** | **Porcine deltacoronavirus** |
| **MF642325.1** | **2017/3/1** | **China** | **Swine** | **Porcine deltacoronavirus** |
| **MF737355.1** | **2001/7/1** | **South Korea** | **Swine** | **Porcine epidemic diarrhea virus** |
| **MF769417.1** | **2017** | **China** | **Swine** | **Swine acute diarrhea syndrome coronavirus** |
| **MF769418.1** | **2017** | **China** | **Swine** | **Swine acute diarrhea syndrome coronavirus** |
| **MF769419.1** | **2017** | **China** | **Swine** | **Swine acute diarrhea syndrome coronavirus** |
| **MF769420.1** | **2017** | **China** | **Swine** | **Swine acute diarrhea syndrome coronavirus** |
| **MF769424.1** | **2017** | **China** | **Swine** | **Swine acute diarrhea syndrome coronavirus** |
| **MF769428.1** | **2017** | **China** | **Swine** | **Swine acute diarrhea syndrome coronavirus** |
| **MF769441.1** | **2017** | **China** | **Swine** | **Swine acute diarrhea syndrome coronavirus** |
| **MF782686.1** | **2015** | **China** | **Swine** | **Porcine epidemic diarrhea virus** |
| **MF782687.1** | **2015** | **China** | **Swine** | **Porcine epidemic diarrhea virus** |
| **MF807951.1** | **2017/2/19** | **China** | **Swine** | **Porcine epidemic diarrhea virus** |
| **MF807952.1** | **2017/2/17** | **China** | **Swine** | **Porcine epidemic diarrhea virus** |
| **MF948005.1** | **2017/8/10** | **China** | **Swine** | **Porcine deltacoronavirus** |
| **MG242062.1** | **2017** | **China** | **Swine** | **Porcine deltacoronavirus** |
| **MG334554.1** | **2017/2/7** | **USA** | **Swine** | **Porcine epidemic diarrhea virus** |
| **MG334555.1** | **2017/2/7** | **USA** | **Swine** | **Porcine epidemic diarrhea virus** |
| **MG546687.1** | **2015/9/1** | **China** | **Swine** | **Porcine epidemic diarrhea virus** |
| **MG546688.1** | **2016/2/1** | **China** | **Swine** | **Porcine epidemic diarrhea virus** |
| **MG546689.1** | **2016/9/1** | **China** | **Swine** | **Porcine epidemic diarrhea virus** |
| **MG546690.1** | **2016/11/1** | **China** | **Swine** | **Porcine epidemic diarrhea virus** |
| **MG557844.1** | **2017/4/1** | **China** | **Swine** | **Swine acute diarrhea syndrome coronavirus** |
| **MG605090.1** | **2017/2/26** | **China** | **Swine** | **Swine acute diarrhea syndrome coronavirus** |
| **MG605091.1** | **2016/8/9** | **China** | **Swine** | **Swine acute diarrhea syndrome coronavirus** |
| **MG742313.1** | **2017/3/14** | **China** | **Swine** | **Porcine enteric alphacoronavirus** |
| **MG781192.1** | **2014/10/23** | **South Korea** | **Swine** | **Porcine epidemic diarrhea virus** |
| **MG832584.1** | **Jul-16** | **China** | **Swine** | **Porcine deltacoronavirus** |
| **MG837011.1** | **2012/2/21** | **China** | **Swine** | **Porcine epidemic diarrhea virus** |
| **MG837012.1** | **2016/1/20** | **China** | **Swine** | **Porcine epidemic diarrhea virus** |
| **MG837058.1** | **2017/5/31** | **USA** | **Swine** | **Porcine epidemic diarrhea virus** |
| **MG837130.1** | **2016/11/1** | **South Korea** | **Swine** | **Porcine deltacoronavirus** |
| **MG837131.1** | **2016/11/1** | **South Korea** | **Swine** | **Porcine deltacoronavirus** |
| **MG983755.1** | **N/A** | **N/A** | **Swine** | **Porcine epidemic diarrhea virus** |
| **MH004412.1** | **2016/7/19** | **Mexico** | **Swine** | **Porcine epidemic diarrhea virus** |
| **MH004413.1** | **2016/12/6** | **Mexico** | **Swine** | **Porcine epidemic diarrhea virus** |
| **MH004414.1** | **2017/5/10** | **Mexico** | **Swine** | **Porcine epidemic diarrhea virus** |
| **MH004415.1** | **2014/7/19** | **Mexico** | **Swine** | **Porcine epidemic diarrhea virus** |
| **MH004416.1** | **2017/2/10** | **Mexico** | **Swine** | **Porcine epidemic diarrhea virus** |
| **MH004417.1** | **2017/2/10** | **Mexico** | **Swine** | **Porcine epidemic diarrhea virus** |
| **MH004418.1** | **2017/2/10** | **Mexico** | **Swine** | **Porcine epidemic diarrhea virus** |
| **MH004419.1** | **2017/2/20** | **Mexico** | **Swine** | **Porcine epidemic diarrhea virus** |
| **MH004420.1** | **2017/2/28** | **Mexico** | **Swine** | **Porcine epidemic diarrhea virus** |
| **MH004421.1** | **2015/11/5** | **Mexico** | **Swine** | **Porcine epidemic diarrhea virus** |
| **MH006957.1** | **2013/7/24** | **Mexico** | **Swine** | **Porcine epidemic diarrhea virus** |
| **MH006958.1** | **2013/9/30** | **Mexico** | **Swine** | **Porcine epidemic diarrhea virus** |
| **MH006959.1** | **2013/9/30** | **Mexico** | **Swine** | **Porcine epidemic diarrhea virus** |
| **MH006960.1** | **2014/11/8** | **Mexico** | **Swine** | **Porcine epidemic diarrhea virus** |
| **MH006961.1** | **2014/7/19** | **Mexico** | **Swine** | **Porcine epidemic diarrhea virus** |
| **MH006962.1** | **2015/3/20** | **Mexico** | **Swine** | **Porcine epidemic diarrhea virus** |
| **MH006963.1** | **2013/3/13** | **Mexico** | **Swine** | **Porcine epidemic diarrhea virus** |
| **MH006964.1** | **2014/7/19** | **Mexico** | **Swine** | **Porcine epidemic diarrhea virus** |
| **MH006965.1** | **2013/7/24** | **Mexico** | **Swine** | **Porcine epidemic diarrhea virus** |
| **MH013462.1** | **2015/9/13** | **Mexico** | **Swine** | **Porcine epidemic diarrhea virus** |
| **MH013463.1** | **2015/10/21** | **Mexico** | **Swine** | **Porcine epidemic diarrhea virus** |
| **MH013464.1** | **2015/10/21** | **Mexico** | **Swine** | **Porcine epidemic diarrhea virus** |
| **MH013465.1** | **2017/6/21** | **Mexico** | **Swine** | **Porcine epidemic diarrhea virus** |
| **MH013466.1** | **2017/6/21** | **Mexico** | **Swine** | **Porcine epidemic diarrhea virus** |
| **MH025762.1** | **2016** | **China** | **Swine** | **Porcine deltacoronavirus** |
| **MH025763.1** | **2016** | **China** | **Swine** | **Porcine deltacoronavirus** |
| **MH025764.1** | **2016** | **China** | **Swine** | **Porcine deltacoronavirus** |
| **MH052681.1** | **2017/3/1** | **South Korea** | **Swine** | **Porcine epidemic diarrhea virus** |
| **MH052682.1** | **2017/7/1** | **South Korea** | **Swine** | **Porcine epidemic diarrhea virus** |
| **MH052683.1** | **2017/11/1** | **South Korea** | **Swine** | **Porcine epidemic diarrhea virus** |
| **MH052684.1** | **2017/12/1** | **South Korea** | **Swine** | **Porcine epidemic diarrhea virus** |
| **MH052685.1** | **2017/12/1** | **South Korea** | **Swine** | **Porcine epidemic diarrhea virus** |
| **MH052687.1** | **2017/6/1** | **South Korea** | **Swine** | **Porcine epidemic diarrhea virus** |
| **MH052688.1** | **2017/5/1** | **South Korea** | **Swine** | **Porcine epidemic diarrhea virus** |
| **MH052689.1** | **2017/4/1** | **South Korea** | **Swine** | **Porcine epidemic diarrhea virus** |
| **MH056657.1** | **N/A** | **N/A** | **Swine** | **Porcine epidemic diarrhea virus** |
| **MH056658.1** | **2014** | **China** | **Swine** | **Porcine epidemic diarrhea virus** |
| **MH061336.1** | **2017/1/5** | **China** | **Swine** | **Porcine epidemic diarrhea virus** |
| **MH061337.1** | **2017/1/5** | **China** | **Swine** | **Porcine epidemic diarrhea virus** |
| **MH061338.1** | **2017/9/9** | **China** | **Swine** | **Porcine epidemic diarrhea virus** |
| **MH061339.1** | **2017/11/27** | **China** | **Swine** | **Porcine epidemic diarrhea virus** |
| **MH061340.1** | **2017/12/1** | **China** | **Swine** | **Porcine epidemic diarrhea virus** |
| **MH061341.1** | **2018/1/12** | **China** | **Swine** | **Porcine epidemic diarrhea virus** |
| **MH061342.1** | **2018/2/6** | **China** | **Swine** | **Porcine epidemic diarrhea virus** |
| **MH061343.1** | **2018/1/29** | **China** | **Swine** | **Porcine epidemic diarrhea virus** |
| **MH107321.1** | **2013/1/16** | **China** | **Swine** | **Porcine epidemic diarrhea virus** |
| **MH107322.1** | **2012/11/5** | **China** | **Swine** | **Porcine epidemic diarrhea virus** |
| **MH117940.1** | **2016/3/1** | **China** | **Swine** | **Porcine epidemic diarrhea virus** |
| **MH118331.1** | **N/A** | **Viet Nam** | **Swine** | **Porcine deltacoronavirus** |
| **MH118332.1** | **N/A** | **Viet Nam** | **Swine** | **Porcine deltacoronavirus** |
| **MH118333.1** | **N/A** | **Viet Nam** | **Swine** | **Porcine deltacoronavirus** |
| **MH243316.1** | **2018/3/1** | **South Korea** | **Swine** | **Porcine epidemic diarrhea virus** |
| **MH243318.1** | **2018/3/1** | **South Korea** | **Swine** | **Porcine epidemic diarrhea virus** |
| **MH243319.1** | **2018/3/1** | **South Korea** | **Swine** | **Porcine epidemic diarrhea virus** |
| **MH539766.1** | **2017/7/1** | **China** | **Swine** | **Porcine enteric alphacoronavirus** |
| **MH581489.1** | **2017/7/20** | **China** | **Swine** | **Porcine epidemic diarrhea virus** |
| **MH593900.1** | **2018/3/20** | **Hungary** | **Swine** | **Porcine epidemic diarrhea virus** |
| **MH615810.1** | **2018/3/1** | **China** | **Swine** | **Swine acute diarrhea syndrome coronavirus** |
| **MH708123.1** | **2018/3/20** | **China** | **Swine** | **Porcine deltacoronavirus** |
| **MH708124.1** | **2018/3/20** | **China** | **Swine** | **Porcine deltacoronavirus** |
| **MH708125.1** | **2018/3/20** | **China** | **Swine** | **Porcine deltacoronavirus** |
| **MH708243.1** | **2017/9/1** | **China** | **Swine** | **Porcine epidemic diarrhea virus** |
| **MH708895.1** | **2011/1/18** | **China** | **Swine** | **Porcine epidemic diarrhea virus** |
| **MH715491.1** | **2016** | **China** | **Swine** | **Porcine deltacoronavirus** |
| **MH726362.1** | **2012/2/8** | **China** | **Swine** | **Porcine epidemic diarrhea virus** |
| **MH726363.1** | **2012/6/22** | **China** | **Swine** | **Porcine epidemic diarrhea virus** |
| **MH726364.1** | **2012/12/13** | **China** | **Swine** | **Porcine epidemic diarrhea virus** |
| **MH726365.1** | **2013/1/15** | **China** | **Swine** | **Porcine epidemic diarrhea virus** |
| **MH726366.1** | **2012/6/4** | **China** | **Swine** | **Porcine epidemic diarrhea virus** |
| **MH726367.1** | **2012/9/17** | **China** | **Swine** | **Porcine epidemic diarrhea virus** |
| **MH726368.1** | **2012/9/23** | **China** | **Swine** | **Porcine epidemic diarrhea virus** |
| **MH726369.1** | **2014/8/21** | **China** | **Swine** | **Porcine epidemic diarrhea virus** |
| **MH726370.1** | **2014/3/12** | **China** | **Swine** | **Porcine epidemic diarrhea virus** |
| **MH726371.1** | **2014/1/9** | **China** | **Swine** | **Porcine epidemic diarrhea virus** |
| **MH726372.1** | **2012/12/13** | **China** | **Swine** | **Porcine epidemic diarrhea virus** |
| **MH726373.1** | **2012/6/15** | **China** | **Swine** | **Porcine epidemic diarrhea virus** |
| **MH726374.1** | **2014/6/7** | **China** | **Swine** | **Porcine epidemic diarrhea virus** |
| **MH726375.1** | **2012/6/4** | **China** | **Swine** | **Porcine epidemic diarrhea virus** |
| **MH726376.1** | **2014/3/24** | **China** | **Swine** | **Porcine epidemic diarrhea virus** |
| **MH726377.1** | **2014/7/11** | **China** | **Swine** | **Porcine epidemic diarrhea virus** |
| **MH726378.1** | **2014/7/28** | **China** | **Swine** | **Porcine epidemic diarrhea virus** |
| **MH726379.1** | **2014/7/28** | **China** | **Swine** | **Porcine epidemic diarrhea virus** |
| **MH726380.1** | **2014/6/6** | **China** | **Swine** | **Porcine epidemic diarrhea virus** |
| **MH726381.1** | **2015/5/6** | **China** | **Swine** | **Porcine epidemic diarrhea virus** |
| **MH726382.1** | **2016/5/18** | **China** | **Swine** | **Porcine epidemic diarrhea virus** |
| **MH726383.1** | **2017/2/27** | **China** | **Swine** | **Porcine epidemic diarrhea virus** |
| **MH726384.1** | **2011/2/10** | **China** | **Swine** | **Porcine epidemic diarrhea virus** |
| **MH726385.1** | **2011/2/10** | **China** | **Swine** | **Porcine epidemic diarrhea virus** |
| **MH726386.1** | **2011/5/25** | **China** | **Swine** | **Porcine epidemic diarrhea virus** |
| **MH726387.1** | **2011/2/10** | **China** | **Swine** | **Porcine epidemic diarrhea virus** |
| **MH726388.1** | **2011/2/10** | **China** | **Swine** | **Porcine epidemic diarrhea virus** |
| **MH726389.1** | **2011/2/10** | **China** | **Swine** | **Porcine epidemic diarrhea virus** |
| **MH726390.1** | **2011/2/10** | **China** | **Swine** | **Porcine epidemic diarrhea virus** |
| **MH726391.1** | **2012/7/15** | **China** | **Swine** | **Porcine epidemic diarrhea virus** |
| **MH726392.1** | **2013/7/2** | **China** | **Swine** | **Porcine epidemic diarrhea virus** |
| **MH726393.1** | **2011/3/10** | **China** | **Swine** | **Porcine epidemic diarrhea virus** |
| **MH726394.1** | **2015/12/8** | **China** | **Swine** | **Porcine epidemic diarrhea virus** |
| **MH726395.1** | **2013/2/6** | **China** | **Swine** | **Porcine epidemic diarrhea virus** |
| **MH726396.1** | **2013/12/7** | **China** | **Swine** | **Porcine epidemic diarrhea virus** |
| **MH726397.1** | **2012/10/20** | **China** | **Swine** | **Porcine epidemic diarrhea virus** |
| **MH726398.1** | **2011/5/25** | **China** | **Swine** | **Porcine epidemic diarrhea virus** |
| **MH726399.1** | **2011/9/25** | **China** | **Swine** | **Porcine epidemic diarrhea virus** |
| **MH726400.1** | **2011/4/26** | **China** | **Swine** | **Porcine epidemic diarrhea virus** |
| **MH726401.1** | **2011/11/27** | **China** | **Swine** | **Porcine epidemic diarrhea virus** |
| **MH726402.1** | **2016/5/6** | **China** | **Swine** | **Porcine epidemic diarrhea virus** |
| **MH726403.1** | **2017/2/24** | **China** | **Swine** | **Porcine epidemic diarrhea virus** |
| **MH726404.1** | **2017/12/17** | **China** | **Swine** | **Porcine epidemic diarrhea virus** |
| **MH726405.1** | **2016/12/6** | **China** | **Swine** | **Porcine epidemic diarrhea virus** |
| **MH726406.1** | **2017/2/25** | **China** | **Swine** | **Porcine epidemic diarrhea virus** |
| **MH726407.1** | **2017/2/25** | **China** | **Swine** | **Porcine epidemic diarrhea virus** |
| **MH726408.1** | **2014/8/1** | **China** | **Swine** | **Porcine epidemic diarrhea virus** |
| **MH748550.1** | **2017/11/10** | **China** | **Swine** | **Porcine epidemic diarrhea virus** |
| **MH891584.1** | **2018/2/6** | **South Korea** | **Swine** | **Porcine epidemic diarrhea virus** |
| **MH891585.1** | **2018/2/7** | **South Korea** | **Swine** | **Porcine epidemic diarrhea virus** |
| **MH891586.1** | **2018/4/18** | **South Korea** | **Swine** | **Porcine epidemic diarrhea virus** |
| **MH891587.1** | **2018/4/19** | **South Korea** | **Swine** | **Porcine epidemic diarrhea virus** |
| **MH891588.1** | **2017/2/21** | **South Korea** | **Swine** | **Porcine epidemic diarrhea virus** |
| **MH891589.1** | **2018/1/19** | **South Korea** | **Swine** | **Porcine epidemic diarrhea virus** |
| **MH891590.1** | **2018/2/6** | **South Korea** | **Swine** | **Porcine epidemic diarrhea virus** |
| **MH910099.1** | **2013/1/2** | **China** | **Swine** | **Porcine epidemic diarrhea virus** |
| **MK005882.1** | **2018/3/1** | **China** | **Swine** | **Porcine deltacoronavirus** |
| **MK032689.1** | **2018/3/1** | **South Korea** | **Swine** | **Porcine epidemic diarrhea virus** |
| **MK032690.1** | **2018/4/1** | **South Korea** | **Swine** | **Porcine epidemic diarrhea virus** |
| **MK032691.1** | **2018/4/1** | **South Korea** | **Swine** | **Porcine epidemic diarrhea virus** |
| **MK032692.1** | **2018/4/1** | **South Korea** | **Swine** | **Porcine epidemic diarrhea virus** |
| **MK071619.1** | **2015/1/9** | **Colombia** | **Swine** | **Porcine epidemic diarrhea virus** |
| **MK071620.1** | **2015/1/29** | **Colombia** | **Swine** | **Porcine epidemic diarrhea virus** |
| **MK071621.1** | **2014/3/7** | **Colombia** | **Swine** | **Porcine epidemic diarrhea virus** |
| **MK071622.1** | **2014/3/17** | **Colombia** | **Swine** | **Porcine epidemic diarrhea virus** |
| **MK071623.1** | **2014/3/16** | **Colombia** | **Swine** | **Porcine epidemic diarrhea virus** |
| **MK071624.1** | **2014/3/21** | **Colombia** | **Swine** | **Porcine epidemic diarrhea virus** |
| **MK071625.1** | **2015/4/27** | **Colombia** | **Swine** | **Porcine epidemic diarrhea virus** |
| **MK071626.1** | **2015/5/6** | **Colombia** | **Swine** | **Porcine epidemic diarrhea virus** |
| **MK071627.1** | **2015/5/8** | **Colombia** | **Swine** | **Porcine epidemic diarrhea virus** |
| **MK071628.1** | **2015/5/20** | **Colombia** | **Swine** | **Porcine epidemic diarrhea virus** |
| **MK071629.1** | **2015/5/20** | **Colombia** | **Swine** | **Porcine epidemic diarrhea virus** |
| **MK071630.1** | **2015/6/3** | **Colombia** | **Swine** | **Porcine epidemic diarrhea virus** |
| **MK071631.1** | **2015/1/6** | **Colombia** | **Swine** | **Porcine epidemic diarrhea virus** |
| **MK071632.1** | **2017/7/1** | **Colombia** | **Swine** | **Porcine epidemic diarrhea virus** |
| **MK071633.1** | **2015/7/3** | **Colombia** | **Swine** | **Porcine epidemic diarrhea virus** |
| **MK071634.1** | **2015/7/3** | **Colombia** | **Swine** | **Porcine epidemic diarrhea virus** |
| **MK071635.1** | **2015/7/30** | **Colombia** | **Swine** | **Porcine epidemic diarrhea virus** |
| **MK071636.1** | **2015/7/29** | **Colombia** | **Swine** | **Porcine epidemic diarrhea virus** |
| **MK071637.1** | **2015/8/11** | **Colombia** | **Swine** | **Porcine epidemic diarrhea virus** |
| **MK071638.1** | **2015/10/21** | **Colombia** | **Swine** | **Porcine epidemic diarrhea virus** |
| **MK071639.1** | **2015/5/5** | **Colombia** | **Swine** | **Porcine epidemic diarrhea virus** |
| **MK138353.1** | **2016** | **China** | **Swine** | **Porcine epidemic diarrhea virus** |
| **MK138516.1** | **2018/3/9** | **China** | **Swine** | **Porcine epidemic diarrhea virus** |
| **MK140811.1** | **2018** | **China** | **Swine** | **Porcine epidemic diarrhea virus** |
| **MK140812.1** | **2018** | **China** | **Swine** | **Porcine epidemic diarrhea virus** |
| **MK140813.1** | **2018** | **China** | **Swine** | **Porcine epidemic diarrhea virus** |
| **MK140814.1** | **2018** | **China** | **Swine** | **Porcine epidemic diarrhea virus** |
| **MK211169.1** | **2017/12/24** | **China** | **Swine** | **Porcine deltacoronavirus** |
| **MK250953.1** | **2018/2/1** | **China** | **Swine** | **Porcine epidemic diarrhea virus** |
| **MK288006.1** | **2011/5/22** | **China** | **Swine** | **Porcine epidemic diarrhea virus** |
| **MK330604.1** | **2017/2/1** | **China** | **Swine** | **Porcine deltacoronavirus** |
| **MK330605.1** | **2018/1/1** | **China** | **Swine** | **Porcine deltacoronavirus** |
| **MK355396.1** | **2016/2/20** | **China** | **Swine** | **Porcine deltacoronavirus** |
| **MK359104.1** | **2018** | **China** | **Swine** | **Porcine deltacoronavirus** |
| **MK392335.1** | **2010/2/1** | **China** | **Swine** | **Porcine epidemic diarrhea virus** |
| **MK409657.1** | **2016/1/31** | **China** | **Swine** | **Porcine epidemic diarrhea virus** |
| **MK409658.1** | **2016/2/23** | **China** | **Swine** | **Porcine epidemic diarrhea virus** |
| **MK409659.1** | **2016/6/16** | **China** | **Swine** | **Porcine epidemic diarrhea virus** |
| **MK482396.1** | **2018/11/1** | **South Korea** | **Swine** | **Porcine epidemic diarrhea virus** |
| **MK482397.1** | **2018/11/1** | **South Korea** | **Swine** | **Porcine epidemic diarrhea virus** |
| **MK558089.1** | **2017/4/4** | **Philippines** | **Swine** | **Porcine epidemic diarrhea virus** |
| **MK559454.1** | **2014** | **South Korea** | **Swine** | **Porcine epidemic diarrhea virus** |
| **MK559455.1** | **2014** | **South Korea** | **Swine** | **Porcine epidemic diarrhea virus** |
| **MK559456.1** | **2018** | **South Korea** | **Swine** | **Porcine epidemic diarrhea virus** |
| **MK572803.1** | **2017/6/1** | **China** | **Swine** | **Porcine deltacoronavirus** |
| **MK584552.1** | **2011/5/1** | **China** | **Swine** | **Porcine epidemic diarrhea virus** |
| **MK606368.1** | **2018/6/24** | **China** | **Swine** | **Porcine epidemic diarrhea virus** |
| **MK606369.1** | **2018/8/6** | **China** | **Swine** | **Porcine epidemic diarrhea virus** |
| **MK625638.1** | **2018/10/1** | **China** | **Swine** | **Porcine deltacoronavirus** |
| **MK625639.1** | **2018/10/1** | **China** | **Swine** | **Porcine deltacoronavirus** |
| **MK625640.1** | **2018/10/1** | **China** | **Swine** | **Porcine deltacoronavirus** |
| **MK625641.1** | **2018/10/1** | **China** | **Swine** | **Porcine deltacoronavirus** |
| **MK644601.1** | **2017/6/1** | **China** | **Swine** | **Porcine epidemic diarrhea virus** |
| **MK644602.1** | **2017/10/20** | **China** | **Swine** | **Porcine epidemic diarrhea virus** |
| **MK644603.1** | **2017/10/27** | **China** | **Swine** | **Porcine epidemic diarrhea virus** |
| **MK644604.1** | **2017/11/13** | **China** | **Swine** | **Porcine epidemic diarrhea virus** |
| **MK644605.1** | **2018/2/2** | **China** | **Swine** | **Porcine epidemic diarrhea virus** |
| **MK651076.1** | **2019/2/1** | **China** | **Swine** | **Swine acute diarrhea syndrome coronavirus** |
| **MK673545.1** | **2018/2/27** | **Taiwan** | **Swine** | **Porcine epidemic diarrhea virus** |
| **MK690502.1** | **2016/3/1** | **China** | **Swine** | **Porcine epidemic diarrhea virus** |
| **MK702008.1** | **2018/10/1** | **China** | **Swine** | **Porcine epidemic diarrhea virus** |
| **MK796238.1** | **2018/3/1** | **China** | **Swine** | **Porcine epidemic diarrhea virus** |
| **MK841494.1** | **2016/10/1** | **China** | **Swine** | **Porcine epidemic diarrhea virus** |
| **MK841495.1** | **2016/9/1** | **China** | **Swine** | **Porcine epidemic diarrhea virus** |
| **MK862249.1** | **2017/8/1** | **China** | **Swine** | **Porcine epidemic diarrhea virus** |
| **MK977618.1** | **2018** | **China** | **Swine** | **Swine enteric alphacoronavirus** |
| **MK993519.1** | **2019/1/1** | **China** | **Swine** | **Porcine deltacoronavirus** |
| **MK994934.1** | **2017** | **China** | **Swine** | **Swine acute diarrhea syndrome coronavirus** |
| **MK994935.1** | **2018/3/1** | **China** | **Swine** | **Swine acute diarrhea syndrome coronavirus** |
| **MK994936.1** | **2018/7/1** | **China** | **Swine** | **Swine acute diarrhea syndrome coronavirus** |
| **MK994937.1** | **2018/10/1** | **China** | **Swine** | **Swine acute diarrhea syndrome coronavirus** |
| **MN025260.1** | **2017/1/18** | **China** | **Swine** | **Porcine deltacoronavirus** |
| **MN037494.1** | **2017/4/18** | **China** | **Swine** | **Porcine epidemic diarrhea virus** |
| **MN056942.1** | **2019/2/1** | **France** | **Swine** | **Porcine epidemic diarrhea virus** |
| **MN114121.1** | **2018/3/1** | **China** | **Swine** | **Porcine epidemic diarrhea virus** |
| **MN173779.1** | **2018** | **China** | **Swine** | **Porcine deltacoronavirus** |
| **MN173780.1** | **2018** | **China** | **Swine** | **Porcine deltacoronavirus** |
| **MN173781.1** | **2018** | **China** | **Swine** | **Porcine deltacoronavirus** |
| **MN173782.1** | **2018** | **China** | **Swine** | **Porcine deltacoronavirus** |
| **MN249445.1** | **2017/12/11** | **China** | **Swine** | **Porcine deltacoronavirus** |
| **MN315264.1** | **2018/9/30** | **China** | **Swine** | **Porcine epidemic diarrhea virus** |
| **MN486588.1** | **2016/2/1** | **China** | **Swine** | **Porcine epidemic diarrhea virus** |
| **MN594506.1** | **2018/3/27** | **China** | **Swine** | **Porcine epidemic diarrhea virus** |
| **MN644470.1** | **2015** | **China** | **Swine** | **Porcine epidemic diarrhea virus** |
| **MN692770.1** | **2014/5/30** | **Spain** | **Swine** | **Swine enteric coronavirus** |
| **MN692771.1** | **2014/4/24** | **Spain** | **Swine** | **Porcine epidemic diarrhea virus** |
| **MN692772.1** | **2014/4/29** | **Spain** | **Swine** | **Porcine epidemic diarrhea virus** |
| **MN692773.1** | **2014/4/29** | **Spain** | **Swine** | **Porcine epidemic diarrhea virus** |
| **MN692774.1** | **2014/6/3** | **Spain** | **Swine** | **Porcine epidemic diarrhea virus** |
| **MN692775.1** | **2014/7/11** | **Spain** | **Swine** | **Porcine epidemic diarrhea virus** |
| **MN692776.1** | **2014/9/30** | **Spain** | **Swine** | **Porcine epidemic diarrhea virus** |
| **MN692777.1** | **2014/9/30** | **Spain** | **Swine** | **Porcine epidemic diarrhea virus** |
| **MN692778.1** | **2014/10/7** | **Spain** | **Swine** | **Porcine epidemic diarrhea virus** |
| **MN692779.1** | **2014/10/9** | **Spain** | **Swine** | **Porcine epidemic diarrhea virus** |
| **MN692780.1** | **2014/12/19** | **Spain** | **Swine** | **Porcine epidemic diarrhea virus** |
| **MN692781.1** | **2016/2/12** | **Spain** | **Swine** | **Porcine epidemic diarrhea virus** |
| **MN692782.1** | **2016/9/23** | **Spain** | **Swine** | **Porcine epidemic diarrhea virus** |
| **MN692783.1** | **2016/12/5** | **Spain** | **Swine** | **Porcine epidemic diarrhea virus** |
| **MN692784.1** | **2017/1/19** | **Spain** | **Swine** | **Porcine epidemic diarrhea virus** |
| **MN692785.1** | **2017/1/19** | **Spain** | **Swine** | **Porcine epidemic diarrhea virus** |
| **MN692786.1** | **2017/1/19** | **Spain** | **Swine** | **Porcine epidemic diarrhea virus** |
| **MN692787.1** | **2018/1/11** | **Spain** | **Swine** | **Porcine epidemic diarrhea virus** |
| **MN692788.1** | **2018/2/2** | **Spain** | **Swine** | **Porcine epidemic diarrhea virus** |
| **MN692789.1** | **2018/3/2** | **Spain** | **Swine** | **Porcine epidemic diarrhea virus** |
| **MN692790.1** | **2018/5/3** | **Spain** | **Swine** | **Porcine epidemic diarrhea virus** |
| **MN692791.1** | **2019/1/30** | **Spain** | **Swine** | **Porcine epidemic diarrhea virus** |
| **MN692792.1** | **2019/1/7** | **Spain** | **Swine** | **Porcine epidemic diarrhea virus** |
| **MN692793.1** | **2019/5/11** | **Spain** | **Swine** | **Porcine epidemic diarrhea virus** |
| **MN759311.1** | **2019/3/18** | **China** | **Swine** | **Porcine epidemic diarrhea virus** |
| **MN781985.1** | **N/A** | **China** | **Swine** | **Porcine deltacoronavirus** |
| **MN816181.1** | **2018/3/1** | **South Korea** | **Swine** | **Porcine epidemic diarrhea virus** |
| **MN841671.1** | **2019/2/1** | **China** | **Swine** | **Porcine epidemic diarrhea virus** |
| **MN844888.1** | **2019/3/1** | **South Korea** | **Swine** | **Porcine epidemic diarrhea virus** |
| **MN942260.1** | **2015** | **China** | **Swine** | **Porcine deltacoronavirus** |
| **MN971595.1** | **2019/1/1** | **South Korea** | **Swine** | **Porcine epidemic diarrhea virus** |
| **MN971596.1** | **2018/2/1** | **South Korea** | **Swine** | **Porcine epidemic diarrhea virus** |
| **MT039231.1** | **N/A** | **N/A** | **Swine** | **Swine acute diarrhea syndrome coronavirus** |
| **MT039232.1** | **N/A** | **N/A** | **Swine** | **Swine acute diarrhea syndrome coronavirus** |
| **MT090145.1** | **2018** | **China** | **Swine** | **Porcine epidemic diarrhea virus** |
| **MT090146.1** | **2018** | **China** | **Swine** | **Porcine epidemic diarrhea virus** |
| **MT166307.1** | **2018/5/1** | **China** | **Swine** | **Porcine epidemic diarrhea virus** |
| **MT198679.1** | **2018/2/2** | **Viet Nam** | **Swine** | **Porcine epidemic diarrhea virus** |
| **MT199591.1** | **2016/4/8** | **China** | **Swine** | **Swine acute diarrhea syndrome coronavirus** |
| **MT199592.1** | **2018/9/9** | **China** | **Swine** | **Swine acute diarrhea syndrome coronavirus** |
| **MT227371.1** | **2019/10/1** | **Peru** | **Swine** | **Porcine deltacoronavirus** |
| **MT260149.1** | **2018/5/25** | **China** | **Swine** | **Porcine deltacoronavirus** |
| **MT260150.1** | **2018/6/5** | **China** | **Swine** | **Porcine deltacoronavirus** |
| **MT263013.1** | **2017** | **China** | **Swine** | **Porcine deltacoronavirus** |
| **MT263014.1** | **2019/10/10** | **China** | **Swine** | **Porcine epidemic diarrhea virus** |
| **MT294722.1** | **2018** | **China** | **Swine** | **Swine acute diarrhea syndrome coronavirus** |
| **MT303066.1** | **2019/2/1** | **China** | **Swine** | **Porcine epidemic diarrhea virus** |
| **MT338517.1** | **N/A** | **China** | **Swine** | **Porcine epidemic diarrhea virus** |
| **MT338518.1** | **N/A** | **China** | **Swine** | **Porcine epidemic diarrhea virus** |
| **MT490315.1** | **2018** | **Mexico** | **Swine** | **Porcine epidemic diarrhea virus** |
| **MT490316.1** | **2018** | **Mexico** | **Swine** | **Porcine epidemic diarrhea virus** |
| **MT547179.1** | **2017/1/1** | **China** | **Swine** | **Porcine epidemic diarrhea virus** |
| **MT547180.1** | **2017/1/1** | **China** | **Swine** | **Porcine epidemic diarrhea virus** |
| **MT576083.1** | **2016/3/1** | **China** | **Swine** | **Transmissible gastroenteritis virus** |
| **MT602520.1** | **2014** | **Spain** | **Swine** | **Porcine epidemic diarrhea virus** |
| **MT683617.1** | **2016/9/1** | **China** | **Swine** | **Porcine epidemic diarrhea virus** |
| **MT747186.1** | **2017/2/1** | **China** | **Swine** | **Swine acute diarrhea syndrome coronavirus** |
| **MT747187.1** | **2017/2/1** | **China** | **Swine** | **Swine acute diarrhea syndrome coronavirus** |
| **MT747188.1** | **2017/2/1** | **China** | **Swine** | **Swine acute diarrhea syndrome coronavirus** |
| **MT843277.1** | **2013/2/1** | **China** | **Swine** | **Porcine epidemic diarrhea virus** |
| **MT843278.1** | **2019/12/1** | **China** | **Swine** | **Porcine epidemic diarrhea virus** |
| **MT843279.1** | **2013/2/1** | **China** | **Swine** | **Porcine epidemic diarrhea virus** |
| **MT843280.1** | **2019/12/1** | **China** | **Swine** | **Porcine epidemic diarrhea virus** |
| **MW165327.1** | **2014/1/21** | **Taiwan** | **Swine** | **Porcine epidemic diarrhea virus** |
| **MW165328.1** | **2014/1/21** | **Taiwan** | **Swine** | **Porcine epidemic diarrhea virus** |
| **MW165329.1** | **2013/7/1** | **Taiwan** | **Swine** | **Porcine epidemic diarrhea virus** |
| **MW196362.1** | **2019** | **USA** | **Swine** | **Porcine deltacoronavirus** |
| **MW685622.1** | **2014/12/15** | **Haiti** | **Homo sapiens** | **Porcine deltacoronavirus** |
| **MW685623.1** | **2015/3/16** | **Haiti** | **Homo sapiens** | **Porcine deltacoronavirus** |
| **MW685624.1** | **2015/4/13** | **Haiti** | **Homo sapiens** | **Porcine deltacoronavirus** |
| **MW727454.1** | **2018/9/1** | **China** | **Swine** | **Porcine enteric alphacoronavirus** |
| **JQ065043.2** | **2010** | **Hong Kong** | **Swine** | **Porcine coronavirus HKU15** |
| **OK078017.1** | **2018** | **China** | **Swine** | **Swine acute diarrhea syndrome coronavirus** |
| **OK078018.1** | **2018** | **China** | **Swine** | **Swine acute diarrhea syndrome coronavirus** |

**Table S2.** **Information of all deltacoronaviruses used in this study.**

| **GenBank ID** | **Collection date** | **Country/Region** | **Host** | **Organism** |
| --- | --- | --- | --- | --- |
| **FJ376619.2** | **2007/1/1** | **Hong Kong** | **Red-whiskered bulbul** | **Bulbul coronavirus HKU11-934** |
| **FJ376621.1** | **2007/1/1** | **Hong Kong** | **Grey-backed thrush** | **Thrush coronavirus HKU12-600** |
| **FJ376622.1** | **2007/4/1** | **Hong Kong** | **White-rumped munia** | **Munia coronavirus HKU13-3514** |
| **JQ065042.2** | **2009** | **Hong Kong** | **Swine** | **Porcine coronavirus HKU15** |
| **JQ065043.2** | **2010** | **Hong Kong** | **Swine** | **Porcine coronavirus HKU15** |
| **JQ065044.1** | **2007** | **Hong Kong** | **White-eye** | **White-eye coronavirus HKU16** |
| **JQ065045.1** | **2007** | **Hong Kong** | **Sparrow** | **Sparrow coronavirus HKU17** |
| **JQ065046.1** | **2007** | **Hong Kong** | **Magpie-robin** | **Magpie-robin coronavirus HKU18** |
| **JQ065047.1** | **2007** | **Hong Kong** | **Night-heron** | **Night heron coronavirus HKU19** |
| **JQ065048.1** | **2008** | **Hong Kong** | **Wigeon** | **Wigeon coronavirus HKU20** |
| **JQ065049.1** | **2007** | **Hong Kong** | **Common moorhen** | **Common moorhen coronavirus HKU21** |
| **KJ462462.1** | **2014/1/31** | **USA** | **Swine** | **Porcine coronavirus HKU15** |
| **KJ481931.1** | **2014/1/4** | **USA** | **Swine** | **Deltacoronavirus PDCoV/USA/Illinois121/2014** |
| **KJ567050.1** | **2014/2/20** | **USA** | **Swine** | **Porcine deltacoronavirus 8734/USA-IA/2014** |
| **KJ569769.1** | **2014/2/13** | **USA** | **Swine** | **Porcine coronavirus HKU15** |
| **KJ584355.1** | **2014/2/12** | **USA** | **Swine** | **Porcine coronavirus HKU15** |
| **KJ584356.1** | **2014/2/20** | **USA** | **Swine** | **Porcine coronavirus HKU15** |
| **KJ584357.1** | **2014/3/7** | **USA** | **Swine** | **Porcine coronavirus HKU15** |
| **KJ584358.1** | **2014/2/18** | **USA** | **Swine** | **Porcine coronavirus HKU15** |
| **KJ584359.1** | **2014/2/21** | **USA** | **Swine** | **Porcine coronavirus HKU15** |
| **KJ601777.1** | **2014/1/8** | **USA** | **Swine** | **Deltacoronavirus PDCoV/USA/Illinois133/2014** |
| **KJ601778.1** | **2014/1/8** | **USA** | **Swine** | **Deltacoronavirus PDCoV/USA/Illinois134/2014** |
| **KJ601779.1** | **2014/1/11** | **USA** | **Swine** | **Deltacoronavirus PDCoV/USA/Illinois136/2014** |
| **KJ601780.1** | **2014/1/26** | **USA** | **Swine** | **Deltacoronavirus PDCoV/USA/Ohio137/2014** |
| **KJ620016.1** | **2014/3/18** | **USA** | **Swine** | **Porcine coronavirus HKU15** |
| **KJ769231.1** | **2014/3/1** | **USA** | **Swine** | **Swine deltacoronavirus OhioCVM1/2014** |
| **KM012168.1** | **2014/3/17** | **USA** | **Swine** | **Porcine coronavirus HKU15** |
| **KM820765.1** | **2014/4/1** | **South Korea** | **Swine** | **Porcine deltacoronavirus KNU14-04** |
| **KP757890.1** | **2004/5/24** | **China** | **Swine** | **Porcine deltacoronavirus** |
| **KP757891.1** | **2014/12/26** | **China** | **Swine** | **Porcine deltacoronavirus** |
| **KP757892.1** | **2014/12/20** | **China** | **Swine** | **Porcine deltacoronavirus** |
| **KP981395.1** | **N/A** | **USA** | **N/A** | **Porcine deltacoronavirus** |
| **KR131621.1** | **2015/3/1** | **China** | **Swine** | **Porcine deltacoronavirus** |
| **KR150443.1** | **2015/3/24** | **USA** | **Swine** | **Porcine deltacoronavirus** |
| **KR265847.1** | **2014/3/6** | **USA** | **Swine** | **Porcine deltacoronavirus** |
| **KR265848.1** | **2014/3/14** | **USA** | **Swine** | **Porcine deltacoronavirus** |
| **KR265849.1** | **2014/4/2** | **USA** | **Swine** | **Porcine deltacoronavirus** |
| **KR265850.1** | **2014/4/2** | **USA** | **Swine** | **Porcine deltacoronavirus** |
| **KR265851.1** | **2014/5/13** | **USA** | **Swine** | **Porcine deltacoronavirus** |
| **KR265852.1** | **2014/4/21** | **USA** | **Swine** | **Porcine deltacoronavirus** |
| **KR265853.1** | **2013/10/14** | **USA** | **Swine** | **Porcine deltacoronavirus** |
| **KR265854.1** | **2014/5/21** | **USA** | **Swine** | **Porcine deltacoronavirus** |
| **KR265855.1** | **2014/5/21** | **USA** | **Swine** | **Porcine deltacoronavirus** |
| **KR265856.1** | **2014/2/23** | **USA** | **Swine** | **Porcine deltacoronavirus** |
| **KR265857.1** | **2014/2/23** | **USA** | **Swine** | **Porcine deltacoronavirus** |
| **KR265858.1** | **2014/5/6** | **USA** | **Swine** | **Porcine deltacoronavirus** |
| **KR265859.1** | **2014/2/11** | **USA** | **Swine** | **Porcine deltacoronavirus** |
| **KR265860.1** | **2014/2/5** | **USA** | **Swine** | **Porcine deltacoronavirus** |
| **KR265861.1** | **2014/2/5** | **USA** | **Swine** | **Porcine deltacoronavirus** |
| **KR265862.1** | **2014/3/26** | **USA** | **Swine** | **Porcine deltacoronavirus** |
| **KR265863.1** | **2014/3/27** | **USA** | **Swine** | **Porcine deltacoronavirus** |
| **KR265864.1** | **2014/3/14** | **USA** | **Swine** | **Porcine deltacoronavirus** |
| **KR265865.1** | **2014/6/5** | **USA** | **Swine** | **Porcine deltacoronavirus** |
| **KT021234.1** | **2015/3/20** | **China** | **Swine** | **Porcine deltacoronavirus** |
| **KT266822.1** | **2012** | **China** | **Swine** | **Porcine deltacoronavirus** |
| **KT336560.1** | **2014/11/24** | **China** | **Swine** | **Porcine deltacoronavirus** |
| **KT381613.1** | **2014/5/7** | **USA** | **Swine** | **Porcine coronavirus HKU15** |
| **KU051641.1** | **2015/6/10** | **Thailand** | **Swine** | **Porcine deltacoronavirus** |
| **KU051649.1** | **2015/6/30** | **Thailand** | **Swine** | **Porcine deltacoronavirus** |
| **KU665558.1** | **2014/6/26** | **China** | **N/A** | **Porcine deltacoronavirus** |
| **KU981059.1** | **2015/2/16** | **China** | **Swine** | **Porcine deltacoronavirus** |
| **KU981060.1** | **2015/4/15** | **China** | **N/A** | **Porcine deltacoronavirus** |
| **KU981061.1** | **2015/7/18** | **China** | **N/A** | **Porcine deltacoronavirus** |
| **KU981062.1** | **2015/12/30** | **China** | **N/A** | **Porcine deltacoronavirus** |
| **KU984334.1** | **2015/11/1** | **Thailand** | **Swine** | **Porcine deltacoronavirus** |
| **KX022602.1** | **2015/10/15** | **USA** | **Swine** | **Porcine deltacoronavirus** |
| **KX022603.1** | **2015/12/18** | **USA** | **Swine** | **Porcine deltacoronavirus** |
| **KX022604.1** | **2015/11/27** | **USA** | **Swine** | **Porcine deltacoronavirus** |
| **KX022605.1** | **2015/12/21** | **USA** | **Swine** | **Porcine deltacoronavirus** |
| **KX118627.1** | **2016/1/20** | **Laos** | **Swine** | **Porcine deltacoronavirus** |
| **KX361343.1** | **2013/2/1** | **Thailand** | **Swine** | **Porcine deltacoronavirus** |
| **KX361344.1** | **2013/3/1** | **Thailand** | **Swine** | **Porcine deltacoronavirus** |
| **KX361345.1** | **2015/12/1** | **Thailand** | **Swine** | **Porcine deltacoronavirus** |
| **KX443143.2** | **2016** | **China** | **N/A** | **Porcine deltacoronavirus** |
| **KX834351.1** | **2015/10/10** | **Viet Nam** | **Swine** | **Porcine deltacoronavirus** |
| **KX834352.1** | **2015/12/8** | **Viet Nam** | **Swine** | **Porcine deltacoronavirus** |
| **KX998969.1** | **2015/12/1** | **Viet Nam** | **Swine** | **Porcine deltacoronavirus** |
| **KY065120.1** | **2016** | **China** | **Swine** | **Porcine deltacoronavirus** |
| **KY293677.1** | **2016/5/23** | **China** | **Swine** | **Porcine deltacoronavirus** |
| **KY293678.1** | **2016/5/23** | **China** | **Swine** | **Porcine deltacoronavirus** |
| **KY354363.1** | **2016/4/1** | **South Korea** | **Swine** | **Porcine deltacoronavirus** |
| **KY354364.1** | **2016/4/1** | **South Korea** | **Swine** | **Porcine deltacoronavirus** |
| **KY363867.1** | **2016/3/18** | **China** | **Swine** | **Porcine deltacoronavirus** |
| **KY363868.1** | **2016/1/5** | **China** | **Swine** | **Porcine deltacoronavirus** |
| **KY364365.1** | **2014/7/1** | **South Korea** | **Swine** | **Porcine deltacoronavirus** |
| **KY513724.1** | **2014** | **China** | **Swine** | **Porcine deltacoronavirus** |
| **KY513725.1** | **2014** | **China** | **Swine** | **Porcine deltacoronavirus** |
| **KY926512.1** | **2016/11/1** | **South Korea** | **Swine** | **Porcine deltacoronavirus** |
| **LC216914.1** | **2014/2/13** | **Hong Kong** | **Swine** | **Coronavirus HKU15** |
| **LC216915.1** | **2014/2/13** | **Hong Kong** | **Swine** | **Coronavirus HKU15** |
| **LC260038.1** | **2014/5/1** | **Japan** | **Swine** | **Porcine deltacoronavirus** |
| **LC260039.1** | **2014/5/1** | **Japan** | **Swine** | **Porcine deltacoronavirus** |
| **LC260040.1** | **2014/5/1** | **Japan** | **Swine** | **Porcine deltacoronavirus** |
| **LC260041.1** | **2014/5/1** | **Japan** | **Swine** | **Porcine deltacoronavirus** |
| **LC260042.1** | **2014/3/1** | **Japan** | **Swine** | **Porcine deltacoronavirus** |
| **LC260043.1** | **2014/8/1** | **Japan** | **Swine** | **Porcine deltacoronavirus** |
| **LC260044.1** | **2014/12/1** | **Japan** | **Swine** | **Porcine deltacoronavirus** |
| **LC260045.1** | **2016/9/1** | **Japan** | **Swine** | **Porcine deltacoronavirus** |
| **MF041982.1** | **2016/12/23** | **China** | **Swine** | **Porcine deltacoronavirus** |
| **MF095123.1** | **2017/2/15** | **China** | **Swine** | **Porcine deltacoronavirus** |
| **MF280390.1** | **2016** | **China** | **Swine** | **Porcine deltacoronavirus** |
| **MF431742.1** | **2015** | **China** | **Swine** | **Porcine deltacoronavirus** |
| **MF431743.1** | **2014** | **China** | **Swine** | **Porcine deltacoronavirus** |
| **MF642322.1** | **2016/8/1** | **China** | **Swine** | **Porcine deltacoronavirus** |
| **MF642323.1** | **2016/8/1** | **China** | **Swine** | **Porcine deltacoronavirus** |
| **MF642324.1** | **2017/4/1** | **China** | **Swine** | **Porcine deltacoronavirus** |
| **MF642325.1** | **2017/3/1** | **China** | **Swine** | **Porcine deltacoronavirus** |
| **MG242062.1** | **2017** | **China** | **Swine** | **Porcine deltacoronavirus** |
| **MG812375.1** | **2017** | **USA** | **Sparrow** | **Sparrow deltacoronavirus** |
| **MG812376.1** | **2017** | **USA** | **Sparrow** | **Sparrow deltacoronavirus** |
| **MG812377.1** | **2017** | **USA** | **Sparrow** | **Sparrow deltacoronavirus** |
| **MG812378.1** | **2017** | **USA** | **Sparrow** | **Sparrow deltacoronavirus** |
| **MG832584.1** | **2016/7/1** | **China** | **Swine** | **Porcine deltacoronavirus** |
| **MG837130.1** | **2016/11/1** | **South Korea** | **Swine** | **Porcine deltacoronavirus** |
| **MG837131.1** | **2016/11/1** | **South Korea** | **Swine** | **Porcine deltacoronavirus** |
| **MG837132.1** | **2016/11/1** | **South Korea** | **Swine** | **Porcine deltacoronavirus** |
| **MG837133.1** | **2016/11/1** | **South Korea** | **Swine** | **Porcine deltacoronavirus** |
| **MH025762.1** | **2016** | **China** | **Swine** | **Porcine deltacoronavirus** |
| **MH025763.1** | **2016** | **China** | **Swine** | **Porcine deltacoronavirus** |
| **MH025764.1** | **2016** | **China** | **Swine** | **Porcine deltacoronavirus** |
| **MH118331.1** | **N/A** | **Viet Nam** | **Swine** | **Porcine deltacoronavirus** |
| **MH118332.1** | **N/A** | **Viet Nam** | **Swine** | **Porcine deltacoronavirus** |
| **MH118333.1** | **N/A** | **Viet Nam** | **Swine** | **Porcine deltacoronavirus** |
| **MH532440.1** | **2015/3/3** | **Poland** | **Coturnix japonica** | **Quail deltacoronavirus** |
| **MH708123.1** | **2018/3/20** | **China** | **Swine** | **Porcine deltacoronavirus** |
| **MH708124.1** | **2018/3/20** | **China** | **Swine** | **Porcine deltacoronavirus** |
| **MH708125.1** | **2018/3/20** | **China** | **Swine** | **Porcine deltacoronavirus** |
| **MH715491.1** | **2016** | **China** | **Swine** | **Porcine deltacoronavirus** |
| **MK005882.1** | **2018/3/1** | **China** | **Swine** | **Porcine deltacoronavirus** |
| **MK211169.1** | **2017/12/24** | **China** | **Swine** | **Porcine deltacoronavirus** |
| **MK330604.1** | **2017/2/1** | **China** | **Swine** | **Porcine deltacoronavirus** |
| **MK330605.1** | **2018/1/1** | **China** | **Swine** | **Porcine deltacoronavirus** |
| **MK355396.1** | **2016/2/20** | **China** | **Swine** | **Porcine deltacoronavirus** |
| **MK359104.1** | **2018** | **China** | **Swine** | **Porcine deltacoronavirus** |
| **MK572803.1** | **2017/6/1** | **China** | **Swine** | **Porcine deltacoronavirus** |
| **MK625638.1** | **2018/10/1** | **China** | **Swine** | **Porcine deltacoronavirus** |
| **MK625639.1** | **2018/10/1** | **China** | **Swine** | **Porcine deltacoronavirus** |
| **MK625640.1** | **2018/10/1** | **China** | **Swine** | **Porcine deltacoronavirus** |
| **MK625641.1** | **2018/10/1** | **China** | **Swine** | **Porcine deltacoronavirus** |
| **MK993519.1** | **2019/1/1** | **China** | **Swine** | **Porcine deltacoronavirus** |
| **MN025260.1** | **2017/1/18** | **China** | **Swine** | **Porcine deltacoronavirus** |
| **MN173779.1** | **2018** | **China** | **N/A** | **Porcine deltacoronavirus** |
| **MN173780.1** | **2018** | **China** | **N/A** | **Porcine deltacoronavirus** |
| **MN173781.1** | **2018** | **China** | **N/A** | **Porcine deltacoronavirus** |
| **MN173782.1** | **2018** | **China** | **N/A** | **Porcine deltacoronavirus** |
| **MN249445.1** | **2017/12/11** | **China** | **Swine** | **Porcine deltacoronavirus** |
| **MN520190.1** | **2018/3/1** | **China** | **N/A** | **Porcine deltacoronavirus** |
| **MN520191.1** | **2019** | **China** | **N/A** | **Porcine deltacoronavirus** |
| **MN520192.1** | **2018/8/1** | **China** | **N/A** | **Porcine deltacoronavirus** |
| **MN520193.1** | **2018** | **China** | **N/A** | **Porcine deltacoronavirus** |
| **MN520194.1** | **2018/10/1** | **China** | **N/A** | **Porcine deltacoronavirus** |
| **MN520195.1** | **2018/3/1** | **China** | **N/A** | **Porcine deltacoronavirus** |
| **MN520196.1** | **2018/3/1** | **China** | **N/A** | **Porcine deltacoronavirus** |
| **MN520197.1** | **2018/4/1** | **China** | **N/A** | **Porcine deltacoronavirus** |
| **MN520198.1** | **2019** | **China** | **N/A** | **Porcine deltacoronavirus** |
| **MN520199.1** | **2018** | **China** | **N/A** | **Porcine deltacoronavirus** |
| **MN520200.1** | **2018** | **China** | **N/A** | **Porcine deltacoronavirus** |
| **MN520201.1** | **2018** | **China** | **N/A** | **Porcine deltacoronavirus** |
| **MN520202.1** | **2018/5/1** | **China** | **N/A** | **Porcine deltacoronavirus** |
| **MN520203.1** | **2018/11/1** | **China** | **N/A** | **Porcine deltacoronavirus** |
| **MN520204.1** | **2018/5/1** | **China** | **N/A** | **Porcine deltacoronavirus** |
| **MN520205.1** | **2019/3/1** | **China** | **N/A** | **Porcine deltacoronavirus** |
| **MN520206.1** | **2019/3/1** | **China** | **N/A** | **Porcine deltacoronavirus** |
| **MN520207.1** | **2018/10/1** | **China** | **N/A** | **Porcine deltacoronavirus** |
| **MN520208.1** | **2018/7/1** | **China** | **N/A** | **Porcine deltacoronavirus** |
| **MN520209.1** | **2019/1/1** | **China** | **N/A** | **Porcine deltacoronavirus** |
| **MN781985.1** | **N/A** | **China** | **Swine** | **Porcine deltacoronavirus** |
| **MN942260.2** | **2015** | **China** | **Swine** | **Porcine deltacoronavirus** |
| **MT138104.1** | **2018** | **N/A** | **N/A** | **Deltacoronavirus sp.** |
| **MT138105.1** | **2018** | **N/A** | **N/A** | **Deltacoronavirus sp.** |
| **MT138108.1** | **2018** | **N/A** | **N/A** | **Deltacoronavirus sp.** |
| **MT227371.1** | **2019/10/1** | **Peru** | **Swine** | **Porcine deltacoronavirus** |
| **MT260149.1** | **2018/5/25** | **China** | **Swine** | **Porcine deltacoronavirus** |
| **MT260150.1** | **2018/6/5** | **China** | **Swine** | **Porcine deltacoronavirus** |
| **MT263013.1** | **2017** | **China** | **Swine** | **Porcine deltacoronavirus** |
| **MT663769.1** | **2019/7/23** | **China** | **Swine** | **Porcine deltacoronavirus** |
| **MW196362.1** | **2019** | **USA** | **Swine** | **Porcine deltacoronavirus** |
| **MW345814.1** | **2018/6/1** | **China** | **Common magpie** | **Deltacoronavirus HNU1-1** |
| **MW345815.1** | **2018/6/1** | **China** | **Common magpie** | **Deltacoronavirus HNU1-2** |
| **MW345816.1** | **2018/6/1** | **China** | **Common magpie** | **Deltacoronavirus HNU3** |
| **MW349841.1** | **2018/6/1** | **China** | **Common magpie** | **Deltacoronavirus HNU2** |
| **MW685622.1** | **2014/12/15** | **Haiti** | **Homo sapiens** | **Porcine deltacoronavirus** |
| **MW685623.1** | **2015/3/16** | **Haiti** | **Homo sapiens** | **Porcine deltacoronavirus** |
| **MW685624.1** | **2015/4/13** | **Haiti** | **Homo sapiens** | **Porcine deltacoronavirus** |
| **MW854634.1** | **2015/6/1** | **Taiwan** | **Swine** | **Porcine deltacoronavirus** |
| **MZ291567.1** | **2014** | **USA** | **Swine** | **Porcine deltacoronavirus** |
| **MZ772936.1** | **2016** | **China** | **Swine** | **Porcine deltacoronavirus** |
| **MZ802772.1** | **2016/9/1** | **Thailand** | **Swine** | **Porcine deltacoronavirus** |
| **MZ802773.1** | **2016/9/1** | **Thailand** | **Swine** | **Porcine deltacoronavirus** |
| **MZ802774.1** | **2016/9/1** | **Thailand** | **Swine** | **Porcine deltacoronavirus** |
| **MZ802775.1** | **2016/1/1** | **Thailand** | **Swine** | **Porcine deltacoronavirus** |
| **MZ802776.1** | **2016/3/1** | **Viet Nam** | **Swine** | **Porcine deltacoronavirus** |
| **MZ802777.1** | **2016/5/1** | **Thailand** | **Swine** | **Porcine deltacoronavirus** |
| **MZ802955.1** | **2020/9/1** | **China** | **Swine** | **Porcine deltacoronavirus** |
| **OK546242.1** | **2020** | **China** | **Swine** | **Porcine deltacoronavirus** |
| **OM256446.1** | **2021/11/1** | **China** | **Swine** | **Porcine deltacoronavirus** |

**Table S3. Information of recombination events detected in swine enteric coronaviruses using RDP4.**

| **Event Number** | **In Alignment** | | **Recombinant Sequence(s)** | **Minor Parental Sequence(s)** | **Major Parental Sequence(s)** | **Detection Methods** | | | | | | |
| --- | --- | --- | --- | --- | --- | --- | --- | --- | --- | --- | --- | --- |
|  | **Begin** | **End** |  |  |  | **RDP** | **GENECONV** | **Bootscan** | **Maxchi** | **Chimaera** | **SiSscan** | **3Seq** |
| **1** | **21510** | **26460** | **MN692770.1 etc.** | **KY019624.1 etc.** | **KT696544.1 etc.** | **NS** | **3.4357E-301** | **NS** | **4.94E-97** | **6.00E-10** | **2E-105** | **3.71E-08** |
| **2** | **25836** | **26142** | **KR061459.1 etc.** | **MF094685.1 etc.** | **DQ201447.1 etc.** | **1.03E-18** | **NS** | **1.59E-09** | **5.69E-08** | **8.62E-34** | **NS** | **1.04E-07** |

**Table S4. Information of recombination events detected in deltacoronaviruses using RDP4.**

| **Event Number** | **In Alignment** | | **Recombinant Sequence(s)** | **Minor Parental Sequence(s)** | **Major Parental Sequence(s)** | **Detection Methods** | | | | | | |
| --- | --- | --- | --- | --- | --- | --- | --- | --- | --- | --- | --- | --- |
|  | **Begin** | **End** |  |  |  | **RDP** | **GENECONV** | **Bootscan** | **Maxchi** | **Chimaera** | **SiSscan** | **3Seq** |
| **1** | **21366** | **24727** | **^FJ376622** | **MH532440** | **MT138108 etc.** | **NS** | **1.13E-19** | **NS** | **2.71E-07** | **1.22E-10** | **1.04E-29** | **2.76E-08** |
| **2** | **22537** | **23543** | **^MH532440** | **MF642324 etc** | **MG812378 etc** | **2.57E-18** | **1.47E-05** | **NS** | **4.30E-04** | **NS** | **1.52E-08** | **2.65E-10** |
| **3** | **24728** | **25634** | **^FJ376622** | **MG812375 etc** | **JQ065046 etc** | **1.40E-36** | **2.32E-04** | **3.43E-29** | **1.52E-03** | **4.92E-16** | **1.48E-14** | **2.65E-10** |
| **4** | **2446** | **6143** | **^MG812377** | **MG812375 etc** | **MG812378** | **NS** | **7.76E-57** | **2.70E-53** | **1.36E-15** | **1.85E-14** | **9.45E-28** | **3.82E-03** |
